# Supplementary material for: The microRNA miR-21 Is a Mediator of FGF8 Action on Cortical COUP-TFI Translation
Source: Stem Cell Reports. 2018 Aug 30;11(3):756–69. doi: 10.1016/j.stemcr.2018.08.002 (PMC6135738; doi:10.1016/j.stemcr.2018.08.002)
Supplement: Document S1. Supplemental Experimental Procedures and Figures S1–S5 [file mmc1.pdf]

Stem Cell Reports, Volume *11*

Supplemental Information

The microRNA *miR-21* Is a Mediator of FGF8 Action on Cortical COUP-TFI Translation

Marco Terrigno, Michele Bertacchi, Luca Pandolfini, Mario Baumgart, Mariantonietta Calvello, Alessandro Cellerino, Michèle Studer, and Federico Cremisi

SUPPLEMENTAL INFORMATION

SUPPLEMENTAL FIGURES

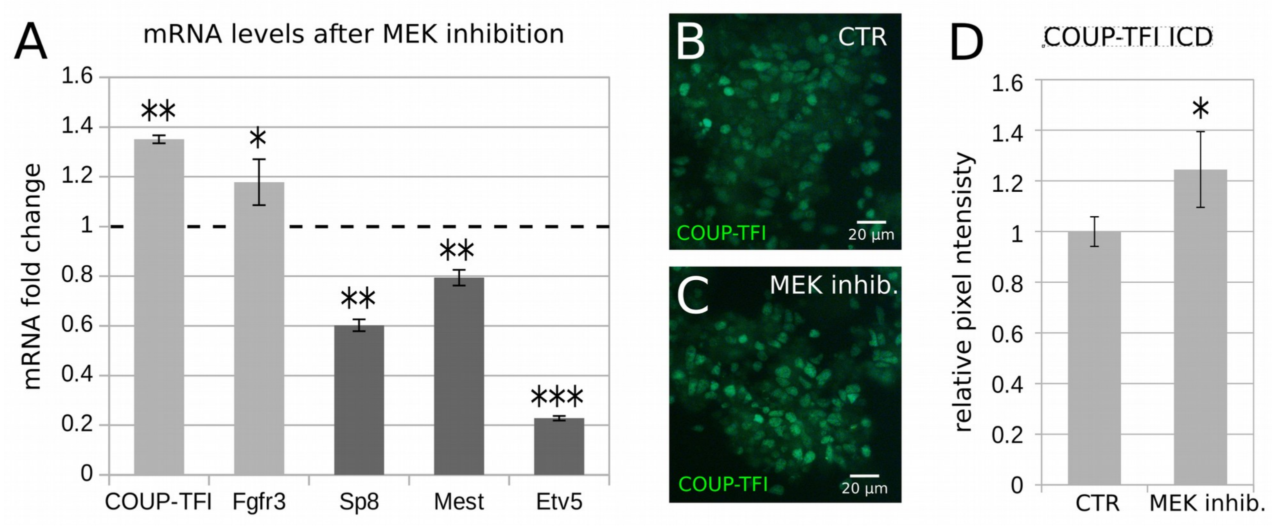

Figure S1 Effect of FGF signaling inhibition on A/P cortical markers and COUP-TFI protein

A, RT-PCR showing fold changes in *COUP-TFI, Fgfr3, Sp8, Mest* and *Etv5* transcript levels at DIV11, after 3 days of FGF signaling inhibition, obtained by means of the MEK inhibitor PD0325901. Error bars represent SEM (n = 3 independent experiments); * $p \leq 0.05$, ** $p \leq 0.01$, *** $p \leq 0.001$, Student T-test. **B-C**, COUP-TFI immunodetection in control DIV11 cells (B), and in DIV11 cells after 3 days of MEK inhibition. **D**, relative pixel intensity of COUP-TFI protein levels in control and MEK-inhibited cells as in B,C. N = 3 independent cultures. Error bars, SEM.

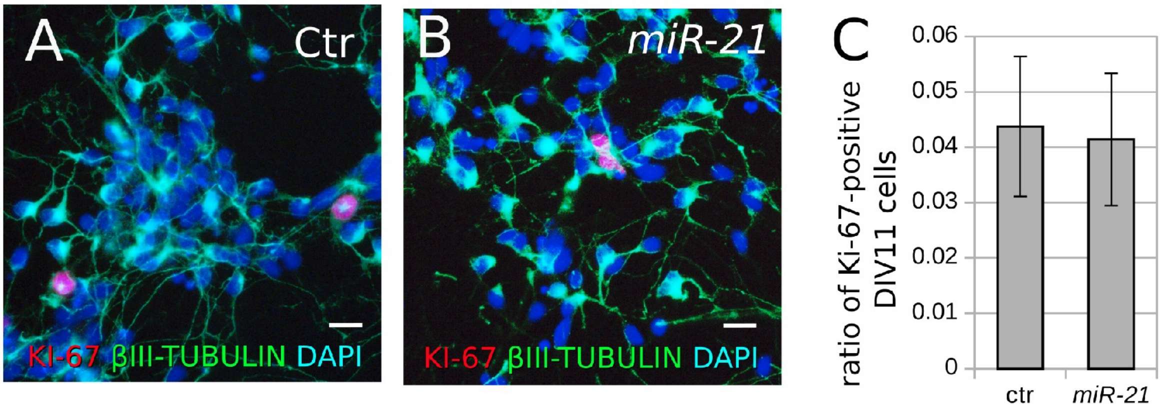

Figure S2 Effect of *miR-21* upregulation on cell proliferation.

A-B, KI-67 (red) and βIII-TUBULIN (green) immunodetection in DIV11 corticalized ES cells lipofected with scrambled (A, Ctr) or *miR-21* (B) mature miRNA at DIV8. Nuclei counterstaining (blue) was obtained with DAPI. Scale bars: 20 μm **C**, Quantification of KI-67- and βIII-TUBULIN-positive cell ratio. N = 3 independent cultures. Error bars, SEM.

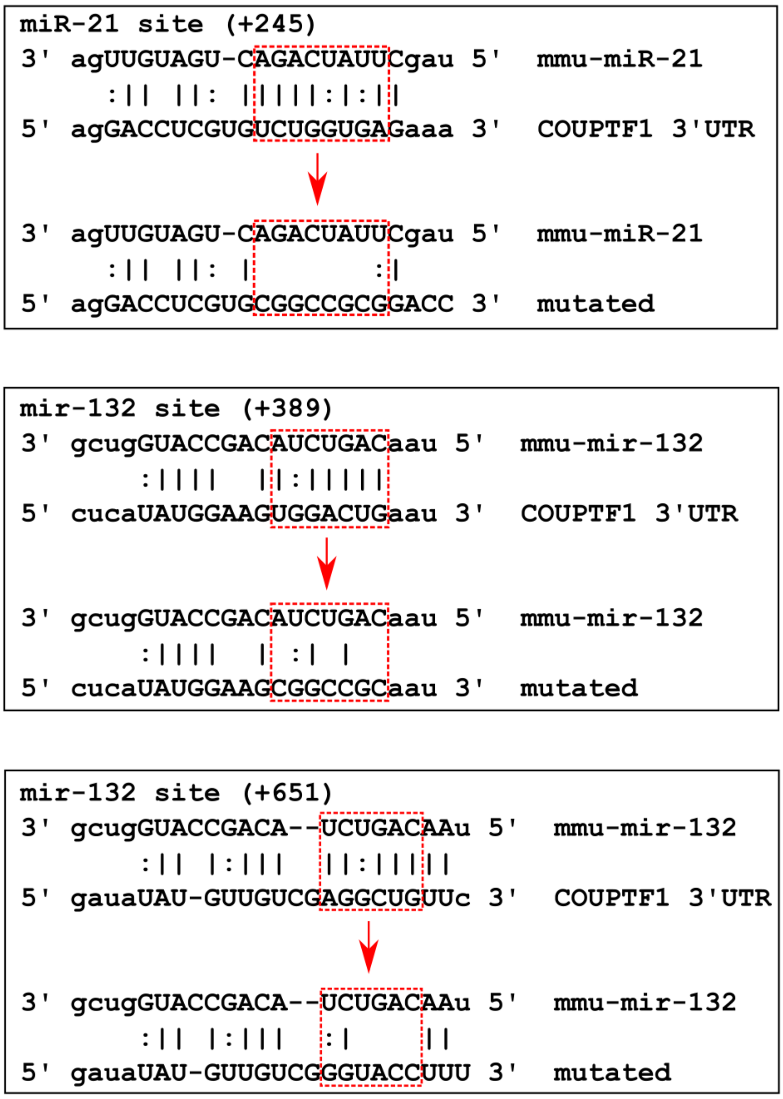

Figure S3 Mutagenesis of the predicted *miR-21* and *miR-132* binding sequences in *COUP-TFI* 3'UTR.

The dashed boxes indicate the seed sequence of miRNA/mRNA interaction predicted by miRANDA. Each seed sequence was mutated to generate distinct EGFP reporter mutants used in Fig. 5F (see Experimental Procedures).

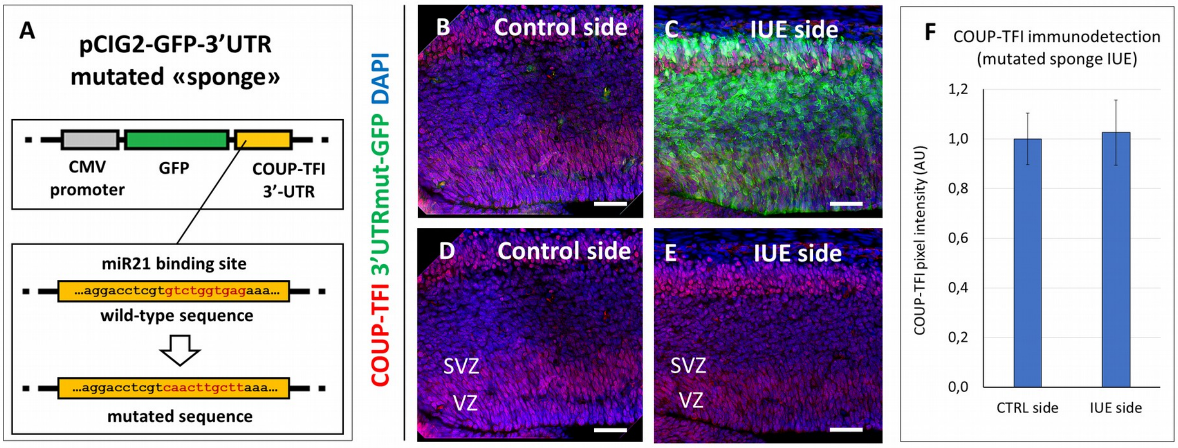

Figure S4 *In vivo* **IUE of** ***miR-21*** **seed-mutated sponge.**

A, Schematics of the control *in utero* electroporation (IUE) experiment using a mutated version of the *COUP-TFI* 3'UTR "sponge" plasmid. The seed sequence of the *miR-21* binding site (GTCTGGTGAG) was replaced with a neutral mutated sequence (CAACTTGCTT; nucleotides highlighted in red). The mutated "sponge" plasmid was injected into the lateral/dorsal pallium of one telencephalic hemisphere and electroporated in neural progenitors, as for experiment in Figure7. Brains were collected 48hr later and processed for immunofluorescence (IF). **B-E**, COUP-TFI IF (red) and GFP (green) in brain sections electroporated with the mutated "sponge" plasmid at E12.5 and analyzed at E14.5. The lateral/dorsal pallium of the electroporated hemisphere (C,E) was compared to the contralateral control hemisphere (B,D). Nuclei counterstaining (blue) was obtained with DAPI. Scale bars: 50 μm. **F**, Pixel intensity quantification (ImageJ) of COUP-TFI fluorescence in electroporated or control cortices (n = 3 brains). The staining was quantified only in the VZ and SVZ. Error bars, SEM.; there was not significant difference between the two conditions (t-test).

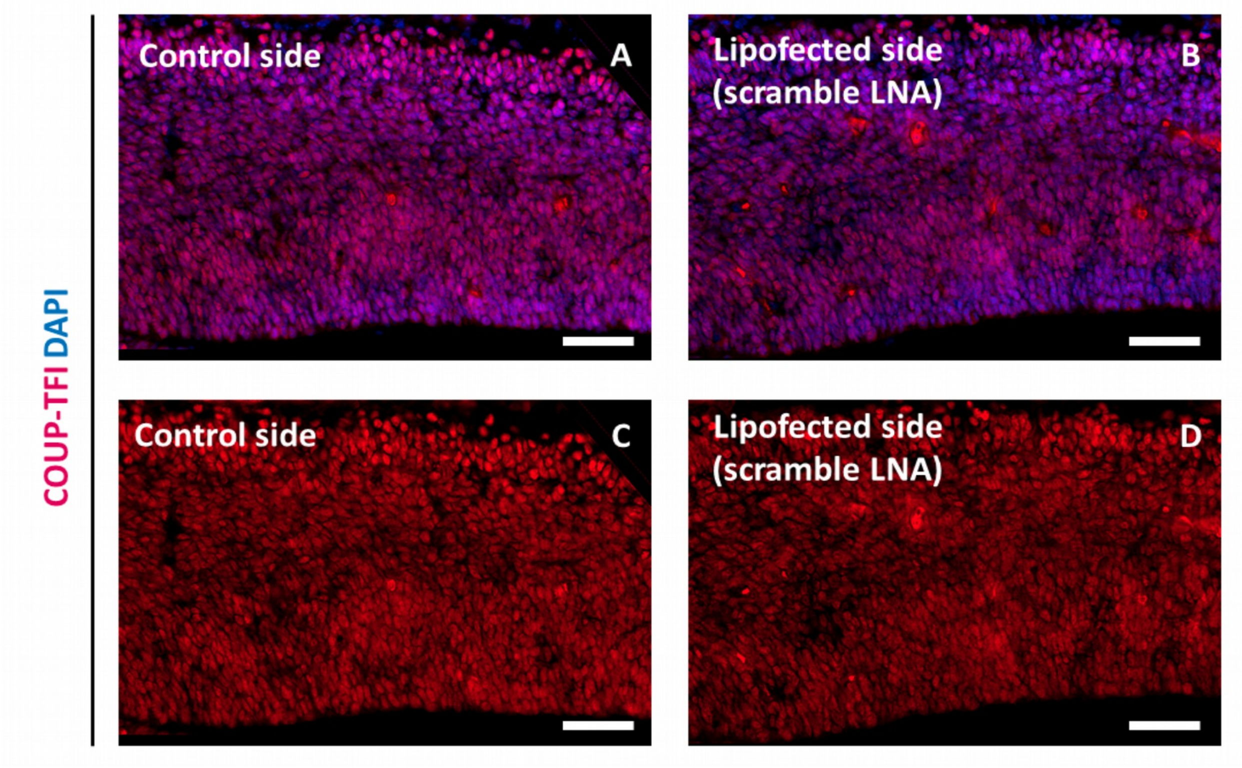

Figure S5 Control LNA lipofection *in vivo*

A-D, COUP-TFI IF (red) in E14.5 brain sections, 2 days after *in utero* lipofection of a scramble (control) LNA. The fluorescence intensity of COUP-TFI protein in neural progenitor regions of the lipofected hemisphere (B,D) was compared to the contralateral control (non-lipofected) hemisphere (A,C). Nuclei counterstaining (blue) was obtained with DAPI. The graph showing pixel intensity quantification corresponding to these images is displayed in Fig 7 L. Scale bar: 50 µm.

SUPPLEMENTAL EXPERIMENTAL PROCEDURES

ICD and RT-PCR

Cells prepared for immunocytodetection experiments were cultured on Poly-ornithine/Laminin coated round glass coverslips. Cells were fixed using 2% paraformaldehyde for 10-15 minutes, washed twice with PBS, permeabilized using 0.1% Triton X100 in PBS and blocked using 0.5% BSA in PBS. Primary antibodies used for ICD included CTIP2 (Abcam ab28448, 1:1000), SATB2 (Abcam ab51502, 1:400), COUP-TFI (Abcam ab60059, 1:1000), KI-67 (ThermoFisher PA5-16446, 1:1000) and TUJ1 (β-III TUBULIN, Covance MRB-435P, 1:1000). Alexa Fluor 488 and Alexa Fluor 546 anti-mouse or anti-rabbit IgG conjugates (ThermoFisher A-11001, A-11008, A-11030 and A-11010, all 1:500) were used as secondary antibodies. Primary antibodies were incubated 2hr at room temperature; cells were then washed three times with PBS (10' each). Alexa Fluor 488 and Alexa Fluor 546 secondary antibodies were incubated 1hr at RT in PBS containing 0.1% Triton X100 and 0.5% BSA for primary antibody detection, followed by three PBS washes (10' each). Nuclear staining was obtained with DAPI.

Total and small RNA was extracted with NucleoSpin RNA II or small RNA columns (Macherey-Nagel). RNA quantity and RNA quality were assessed with Nanodrop and gel electrophoresis. For each sample, 200ng of total RNA was reverse-transcribed, using Eurogentec cDNA synthesis kit for mRNAs and Qiagen miR-script kit for miRNAs. RT-PCR was performed using iTaq Universal SYBR Green Supermix (Bio-rad) on Rotor-Gene 6000 (Corbett). Amplification take-off values were evaluated using the built-in Rotor-Gene 6000 relative quantification analysis function, and relative expression was calculated with the $2^{-\Delta\Delta Ct}$ method, normalizing to the housekeeping gene *β-Actin* or *U6* for mRNAs or miRNAs, respectively. Standard errors were obtained from the error propagation formula, as described (Nordgård et al., 2006). N=3 independent cultures or brain explants were pooled together; each experiment contained n=2 technical replicates.

Reference

Nordgård O, Kvaløy JT, Farmen RK, Heikkilä R. Error propagation in relative real-time reverse transcription polymerase chain reaction quantification models: the balance between accuracy and precision. Anal Biochem. 2006, 356(2):182-93.

List of primers used for RT-PCR

gene	forward sequence	reverse sequence
beta-actin	AATCGTGCGTGACATCAAAG	AAGGAAGGCTGGAAAAGAGC
coupTFI	TGCTTGTGGCCTTGCGGATG	AGTTGCTCGATGACAGAGGAG
coupTFII	TCAACTGCCACTCGTACCTG	CCATGATGTTGTTAGGCTGCAT
efr1	ACGCGACACTACATTCCCC	CTCTTCCGTGTTACGAAGGGC
emx2	GGCTAGAGCACGCTTTTGAG	CACCGGTTAATGTGGTGTGT
en1	AGTGGCGGTGGTAGTGGA	CCTTCTCGTTCTTTTTCTTCTT
epha3	TTCTCCATCTCCGGTGAAAACA	ACCTCCCGACCAGAACATAGG
etv5	TCAGTCTGATAACTTGGTGCTTC	GGCTTCCTATCGTAGGCACAA
fgf15	ATGGCGAGAAAGTGGAACGG	CTGACACAGACTGGGATTGCT
fgf18	CCTGCACTTGCCTGTGTTTAC	TGCTTCCGACTCACATCATCT
fgf8	AGGGGAAGCTAATTGCCAAGA	CCTTGCGGGTAAAGGCCAT
fgfr3	GCCTGCGTGCTAGTGTTCT	TACCATCCTTAGCCCAGACCG
foxg1	CGACCCTGCCCTGTGAGT	TGGAAGAAGACCCCTGATTTTG
hey1	GCGCGGACGAGAATGGAAA	TCAGGTGATCCACAGTCATCTG
Id3	CTGTCGGAACGTAGCCTGG	GTGGTTCATGTCGTCCAAGAG
klf3	GAAGCCCAACAAATATGGGGT	GGACGGGAACTTCAGAGAGG
krox20	GCCAAGGCCGTAGACAAAATC	CCACTCCGTTCATCTGGTCA
mest	GTGGTGGGTCCAAGTAGGG	AAGCACAACTATCTCAGGGCT
otx2	CCACTTCGGGTATGGACTTG	GGTCTTGGCAAACAGAGCTT
pax6	CTTTGCTTGGGAAATCCGAG	AGCCAGGTTGCGAAGAACTC
PTN	ATGTCGTCCCAGCAATATCAGC	CCAAGATGAAAATCAATGCCAGG
sfrp1	CAACGTGGGCTACAAGAAGAT	GGCCAGTAGAAGCCGAAGAAC
sp8	CTGGTAGAGGGTAAGGGCGG	AAGATTGGAAAGGGGGTAGTGA
spry2	TCCAATGACGATGAGGACAA	CACCCCTGGCACAATTTAAG
