# Supplementary material for: The microRNA miR-21 Is a Mediator of FGF8 Action on Cortical COUP-TFI Translation
Source: Stem Cell Reports. 2018 Aug 30;11(3):756–69. doi: 10.1016/j.stemcr.2018.08.002 (PMC6135738; doi:10.1016/j.stemcr.2018.08.002)
Supplement: Document S2. Article plus Supplemental Information [file mmc4.pdf]

Stem Cell Reports
Article
OPEN ACCESS

The microRNA *miR-21* Is a Mediator of FGF8 Action on Cortical COUP-TFI Translation

Marco Terrigno,[1,6] Michele Bertacchi,[2,6] Luca Pandolfini,[1,5] Mario Baumgart,[3] Mariantonietta Calvello,[1] Alessandro Cellerino,[1,3] Michèle Studer,[2,7] and Federico Cremisi[1,4,7,*]
[1]Scuola Normale Superiore, Piazza dei Cavalieri, 7, Pisa 56126, Italy
[2]Université Côte d'Azur (UCA), CNRS, Inserm, iBV, 06108 Nice, France
[3]Leibniz Institute on Aging - Fritz Lipmann Institute (FLI), Beutenbergstraße 11, 07745 Jena, Germany
[4]Istituto di Biofisica CNR, Via Moruzzi 1, 56124 Pisa, Italy
[5]Present address: The Gurdon Institute and Department of Pathology, University of Cambridge, Tennis Court Road, Cambridge CB2 1QN, UK
[6]Co-first author
[7]Co-senior author
*Correspondence: federico.cremisi@sns.it
https://doi.org/10.1016/j.stemcr.2018.08.002

SUMMARY

The morphogen FGF8 plays a pivotal role in neocortical area patterning through its inhibitory effect on *COUP-TFI/Nr2f1* anterior expression, but its mechanism of action is poorly understood. We established an *in vitro* model of mouse embryonic stem cell corticogenesis in which COUP-TFI protein expression is inhibited by the activation of FGF8 in a time window corresponding to cortical area patterning. Interestingly, overexpression of the *COUP-TFI* 3′UTR reduces the inhibitory effect of FGF8 on COUP-TFI translation. FGF8 induces the expression of few miRNAs targeting *COUP-TFI* 3′UTR *in silico*. We found that the functional inhibition of *miR-21* can effectively counteract the inhibitory effect of FGF8 *in vitro* and regulate COUP-TFI protein levels *in vivo*. Accordingly, *miR-21* expression is complementary to COUP-TFI expression during corticogenesis. These data support a translational control of COUP-TFI gradient expression by FGF8 via *miR-21* and contribute to our understanding of how regionalized expression is established during neocortical area mapping.

INTRODUCTION

Neocortical area patterning is a developmental process generating different positional identities along the antero-posterior (A/P) and medio-lateral (M/L) axes of the dorsal telencephalon in mammals and is achieved by the establishment of expression gradients of key transcription factors, such as *Pax6*, *Sp8*, *Emx2*, and *COUP-TFI* (Greig et al., 2013; Sansom and Livesey, 2009; Alfano and Studer, 2013). The orphan nuclear receptor COUP-TFI plays a pivotal role during area patterning, as its cortical inactivation causes the most severe areal disorganization in postnatal neocortices described so far, leading to a massive expansion of the primary motor area at the expense of the somatosensory cortex (Armentano et al., 2007; Alfano et al., 2014).

Fibroblast growth factor (FGF) 8 is a diffusible morphogen acting as a key organizer of the early neocortical area map (Storm, 2006; Fukuchi-Shimogori and Grove, 2001; Assimacopoulos et al., 2012). FGF8 forms an anterior to posterior gradient by diffusing across the mouse neocortical primordium from a discrete source in the rostromedial telencephalon (Toyoda et al., 2010). FGF8 regulates area identity by acting on downstream area mapping genes expressed in progenitors (Sansom et al., 2005; Assimacopoulos et al., 2012). While FGF8 induces *Sp8* expression anteriorly, it also downregulates *Emx2* and *COUP-TFI* expression, which would otherwise promote the development of posterior neocortical identity (Borello et al., 2014; Sansom et al., 2005; Alfano and Studer, 2013). However, among the known mechanisms that mediate FGF8 signaling, alteration of the COUP-TFI gradient has the most striking consequences for area patterning (Bertacchi et al., 2018). A crucial role of FGF8 is thus to repress *COUP-TFI* rostrally by establishing a low anterior to high posterior expression gradient in the neocortical primordium (Garel et al., 2003; Storm, 2006; Alfano and Studer, 2013), but the molecular mechanisms of this inhibition are still not well understood.

There is growing evidence indicating that neural cells generated *in vitro* by mouse embryonic stem cells (ESCs) acquire distinct positional identities through the fine regulation of specific pathways of intracellular signaling (Van den Ameele et al., 2014; Chuang et al., 2015; Lupo et al., 2014). Neural cells with a global gene expression profile resembling naive embryonic cortical cells can be generated by inhibiting BMP and WNT signaling pathways, which are endogenously active during mouse ESC neuralization (Bertacchi et al., 2013, 2015). Notably, after *in vivo* transplantation, these cells exhibit a pattern of connectivity resembling that of their physiological counterparts, suggesting that *in vitro* produced cortical neurons can acquire genuine positional identities (Terrigno et al., 2018).

The opportunity of reproducing *in vitro* the developmental repertoire of neural progenitor cells giving rise to different cortical areas offers a unique tool for studying the molecular signals regulating key genes of area

This is an open access article under the CC BY license (http://creativecommons.org/licenses/by/4.0/).

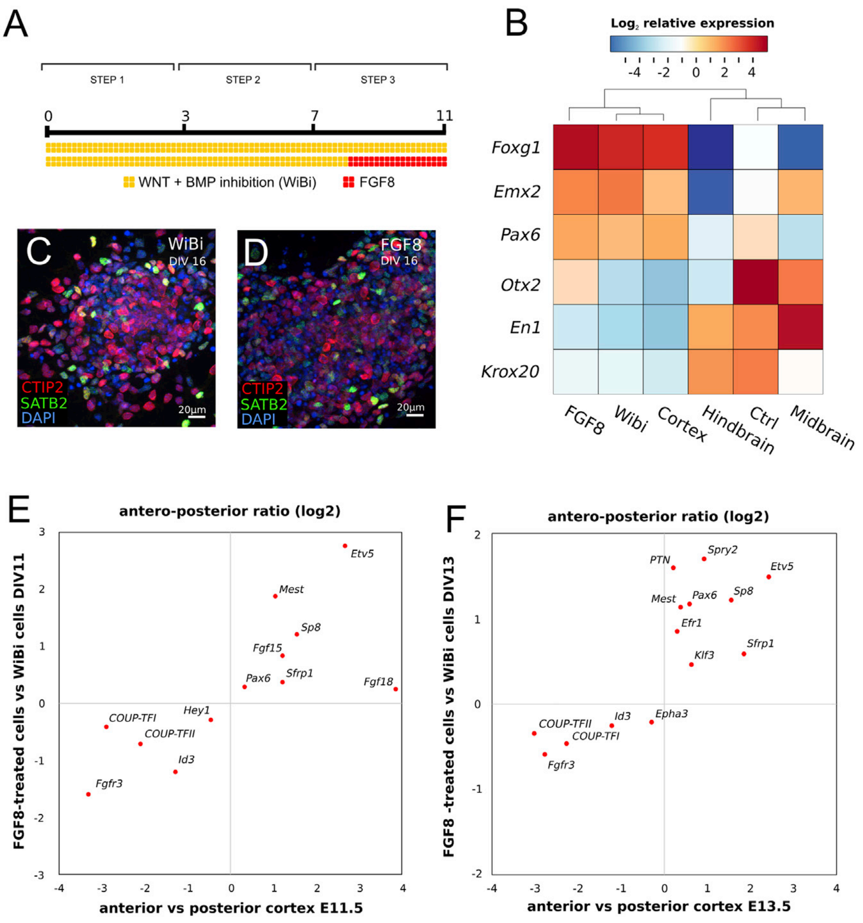

Figure 1. FGF8 Induces Anterior Cortical Identity *In Vitro*

(A) Differentiation protocol with WNT and BMP double inhibition (WiBi, yellow) and FGF8-treatment (FGF8, red).

(B) Color heatmap showing hierarchical clustering and mRNA relative fold change of A/P neural markers, evaluated via RT-PCR, in mouse ESC-derived neurons (WiBi, FGF8, and Ctrl) at DIV11 and in different regions of the E16 embryonic mouse brain (cortex, hindbrain, midbrain). Ctrl, mouse ESCs neuralized without WiBi. Color map shows log2 mean-centered expression. n = 3 independent experiments were pooled together and analyzed by RT-PCR; each experiment contained n = 2 *in vitro* technical replicates.

(C and D) Immunocytodetection (ICD) of pan-cortical markers CTIP2 (red) and SATB2 (green) in WiBi (C) and FGF8 (D) neurons at DIV16.

(E and F) mRNA fold change biplot of A/P cortical markers at E11.5 (E) or E13.5 (F) in WiBi- versus FGF8-treated cells (vertical axis) and anterior versus posterior mouse fetal cortex (horizontal axis). RT-PCR was performed on RNA from n = 3 independent cultures pooled together, each with two *in vitro* technical replicates.

patterning in a dish. We thus investigated the regulation of COUP-TFI protein and *COUP-TFI* mRNA expression in cultures of ESC-derived neural cells. We first found that COUP-TFI protein levels are inhibited by the administration of FGF8, thus indicating the persistence of a molecular machinery coupling FGF8 signaling and COUP-TFI expression *in vitro*. Surprisingly, we observed that the inhibition of COUP-TFI levels by FGF8 occurred principally at the translational level and identified two main FGF8-induced microRNAs (miRNAs) targeting the *COUP-TFI* 3′UTR. Finally, one of the *in vitro* characterized miRNAs conserved its ability to inhibit COUP-TFI during cortical development *in vivo*, indicating that our *in vitro* cellular model can be successfully adopted to gain insight into original developmental mechanisms required for proper neocortical area mapping.

RESULTS

FGF8 Regulates Area-Patterning Genes in an *In Vitro* Model of Corticogenesis

While FGF8 downregulates the expression of forebrain genes and induces the expression of posterior markers during early phases of *in vitro* ESC neuralization (Chiba et al., 2005; Hendrickx et al., 2009), the combination of WNT/BMP inhibition on mouse ESC (mESCs) drives neuralized cells toward a dorsal telencephalic identity after the first week of *in vitro* differentiation (Bertacchi et al., 2015; Yao et al., 2017). Within committed dorsal telencephalic cells, FGF8 then exerts an opposite effect, promoting the acquisition of rostral area identity, hence acting as an anteriorizing cortical factor. We exploited the plasticity of our *in vitro* system to evaluate the effect of FGF8 signaling on cortical area mapping genes. Intracellular FGF8 signaling was activated by FGF8 treatment starting from the eighth day of *in vitro* neuralization (DIV8) of mESCs previously corticalized *via* WNT/BMP inhibition (WiBi) (Figure 1A). To confirm that FGF8 signaling was not affecting telencephalic identity, RT-PCR analysis of general markers of A/P identity was performed at DIV11 on WiBi- and FGF8-treated cells (Figure 1B). FGF8-treated cells express high levels of the anterior markers *FoxG1*, *Emx2*, and *Pax6* and low levels of the posterior markers *En1* and *Krox20*, similarly to cells treated only with WiBi and E16 embryonic mouse cortex (Figure 1B; Bertacchi et al., 2013, 2015).

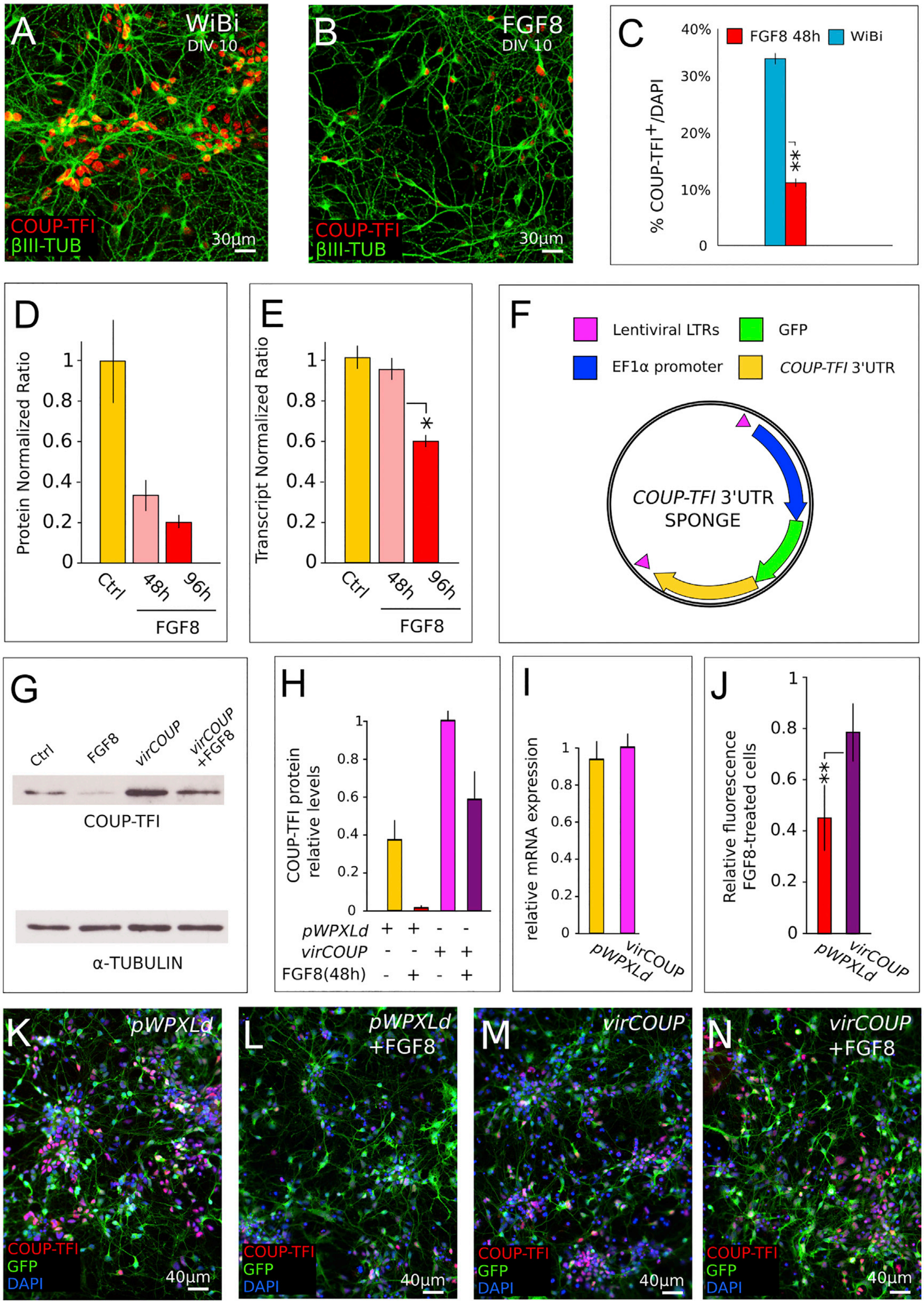

Figure 2. FGF8 Inhibits COUP-TFI Translation Acting on the 3′UTR

(A and B) ICD of COUP-TFI (red) and β-III TUBULIN/TUJI (green) in control cells (A) and after 48 hr of treatment with FGF8 (B) at DIV10.

(legend continued on next page)

Consistently, control cells neuralized in minimal medium and E16 midbrain cells show an opposite trend (Figure 1B). Moreover, activation of FGF8 signaling from DIV8 does not impair the subtype-specific cortical markers CTIP2 and SATB2 (Leone et al., 2008), indicating that the previously acquired cortical identity is not compromised by late FGF8 treatment (Figures 1C and 1D).

Next, we assessed the expression of a broader plethora of genes involved in early regional identity to further evaluate the *in vitro* effect of FGF8 activation on early area patterning. Differentially expressed genes between FGF8- and WiBi-treated cells were correlated to genes differentially expressed between anterior and posterior embryonic cortices (Figures 1E and 1F). Since the cortical neuroepithelium normally shows distinct profiles of genes expressed in gradient at E11.5 (*Hey1*, *Fgf15*, *Fgf18*) and at E13.5 (*Epha3*, *Klf3*, *Efr1*, *Spry2*, *PTN*), we used two separate times of *in vitro* differentiation, DIV11 and DIV13. We found a significant correlation ($r_s = 0.75$; $p < 10^{-5}$) between FGF8-treated cells and embryonic anterior cortex, and between WiBi cells and embryonic posterior cortex, respectively, indicating that FGF8 signaling can regulate the activation of A/P cortical genes *in vitro* similarly to that observed *in vivo*. Finally, the inhibition of endogenous FGF signaling by treating corticalized cells with the MEK inhibitor PD0325901 (Barrett et al., 2008) from DIV8 to DIV11 exerts an opposite effect on A/P cortical markers (Figure S1A), suggesting that endogenous FGF ligands (Bertacchi et al., 2015) might be responsible for modulating A/P markers already in WiBi control cells.

FGF8 Inhibits COUP-TFI Translation by Acting on Its 3′UTR

Among the several FGF8 targets involved in areal patterning, the transcriptional regulator COUP-TFI, a key player of posterior/sensory cortical identity, is inhibited by FGF8 in anterior/motor cortex (Garel et al., 2003; Storm, 2006). We thus assessed whether FGF8 could regulate COUP-TFI also *in vitro* by treating corticalized ESCs either with FGF8 or FGF inhibitor. Notably, FGF8 treated cultures show a marked decrease in the number of cells expressing COUP-TFI (Figures 2A–2C), whereas conversely an increase in COUP-TFI levels is observed in the presence of the FGF inhibitor (Figures S1A–S1D). After 48 hr of FGF8 treatment, we noticed that FGF8 efficiently represses COUP-TFI protein but not *COUP-TFI* transcript levels, which are nevertheless downregulated 96 hr later (Figures 2D and 2E). This indicates that FGF8 might act first and more efficiently on COUP-TFI translation than *COUP-TFI* transcription and suggests a mechanism involving COUP-TFI regulation *via* its 3′UTR.

To directly investigate a possible role of the *COUP-TFI* 3′UTR in inhibiting COUP-TFI translation, we prepared a lentiviral vector (Figure 2F) capable of overexpressing just the 3′UTR (Figures 2G–2N). This construct was transduced into corticalized cells and the expression of COUP-TFI protein (Figures 2G and 2H) and *COUP-TFI* mRNA (Figure 2I) was analyzed 48 hr later. Notably, *COUP-TFI* 3′UTR overexpression reduces the inhibitory effect of FGF8 treatment on COUP-TFI translation, as demonstrated by western blot protein quantifications (Figures 2G and 2H), without affecting transcript levels at the same time point (Figure 2I). Moreover, COUP-TFI protein levels are higher in GFP-expressing cells treated with FGF8 and transduced with the *COUP-TFI* 3′UTR vector when compared with cells treated with FGF8 and transduced with a control vector (Figures 2J–2N). This indicates that *cis*-acting inhibitory signals, located within the 3′UTR, exert their action at cellular levels. Taken together, these data suggest that overexpression of the *COUP-TFI* 3′UTR acts as a "sponge" buffering some *trans*-acting inhibitory signals induced by FGF8.

The FGF8-Induced miRNA *miR-21* Inhibits COUP-TFI Translation *In Vitro*

To investigate whether FGF8 could induce the expression of any miRNAs in corticalized mESCs, we analyzed the

(C) Percentage of COUP-TFI-positive cells in control cells and cells treated with FGF8 for 48 hr. Error bars, SEM; n = 3 independent cultures. $^{**}p \leq 0.01$, Student's t test.
(D) Quantification of COUP-TFI protein levels via western blot analysis after 48 hr and 96 hr of FGF8 treatment.
(E) *COUP-TFI* transcript levels in control and FGF8-treated cells after 48 hr and 96 hr analyzed via RT-PCR. Error bars represent SEM (n = 3 independent experiments). $^{*}p \leq 0.05$, Student's t test.
(F) Schematics of the lentiviral "sponge" vector carrying the *COUP-TFI* 3′UTR.
(G and H) Representative western blot (G) and densitometric analysis (H) of COUP-TFI protein levels in cells transduced with the lentivirus carrying the *COUP-TFI* 3′UTR (*virCOUP*) or the control empty construct (*pWPXLd*) and treated with FGF8 for 48 hr.
(I) *COUP-TFI* transcript levels analyzed via RT-PCR in control cells (*pWPXLd*) or cells overexpressing *COUP-TFI* 3′UTR (*virCOUP*). Error bars represent SEM (n = 3 independent experiments).
(J) Relative COUP-TFI fluorescence intensity (24 hr FGF8-treated versus vehicle) in cells transduced with the *virCOUP* or control vectors; n = 3 independent cultures. Error bars, SEM; $^{**}p \leq 0.01$, Student's t test.
(K–N) ICD of COUP-TFI and GFP in cells transduced with *virCOUP* (M and N) or control vector (K and L) and treated with FGF8 for 24 hr (L and N).
Densitometric analysis in (D) and (H) was performed on n = 2 biological replicas; At least 4–8 wells of 24-well plates were pooled in each replica to compensate for differences in cell seeding.

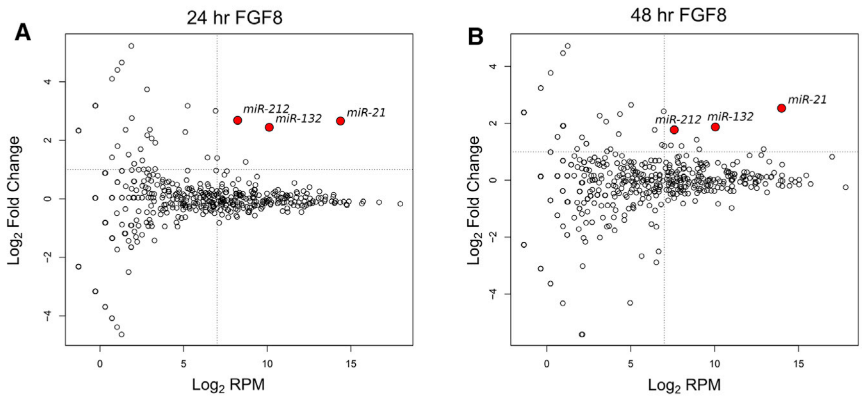

C

24 hr FGF8

miRNA	logFC	logCPM	FDR
miR-132	2.50	10.11	3E-07
miR-212	2.71	8.19	9.7E-07
miR-21	2.74	14.37	0.00211

48 hr FGF8

miRNA	logFC	logCPM	FDR
miR-132	1.86	10.03	8.83E-06
miR-335-5p	1.45	8.92	1.45E-05
miR-21	2.58	14.03	0.00108
miR-212	1.74	7.63	0.0017
miR-148a	1.11	12.91	0.0017
miR-674	1.25	8.03	0.0060
miR-335-3p	1.22	7.4	0.0182

D

```
3' agUUGUAGU-CAGACUAUUCgau 5'    mmu-miR-21       Seed Score: 103
      :|| ||: |||||:|:||
5' agGACCUCGUGUCUGGUGAGaaa 3'    COUPTF1 3'UTR    Energy: -19Kcal/mol

3' gcugGUACCGACA--UCUGACAAu 5'   mmu-miR-132      Seed Score: 139
       :|| |:|||  ||:|||||
5' gauaUAU-GUUGUCGAGGCUGUUc 3'   COUPTF1 3'UTR    Energy: -15,3Kcal/mol

3' gcugGUACCGACAUCUGACaau 5'     mmu-miR-132      Seed Score: 126
        :||||  ||:|||||
5' cucaUAUGGAAGUGGACUGaau 3'     COUPTF1 3'UTR    Energy: -20,3Kcal/mol

3' acCGGCACUGACCU-CUGACaau 5'    mmu-miR-212      Seed Score: 96
      | || ||  ||| |:|||
5' caGACGGGA--GGAGGGCUGgga 3'    COUPTF1 3'UTR    Energy: -15,4Kcal/mol
```

Figure 3. FGF8 Activates miRNAs Targeting the *COUP-TFI* 3′UTR *In Silico*

(A and B) Biplot showing the mean expression levels (log2 RPM) versus fold change (log2 FC) for each microRNA detected, measured after 24 hr (A) or 48 hr (B) of FGF8 treatment.

(C) Lists of the miRNAs significantly enriched after 24 hr and 48 hr of FGF8 treatment. Labeled in red are the miRNAs predicted to bind the *COUP-TFI* 3′UTR and significantly enriched at both time points.

(D) Putative binding sites of miRNAs predicted to target the *COUP-TFI* 3′UTR. "Seed score" and "binding energy" were evaluated using the miRanda algorithm (see Experimental Procedures).

miRNA global expression profiles of cells after 24 or 48 hr of FGF8 treatment (Table S1). After 24 hr, only a few miRNAs show a significant increased expression (false discovery rate <0.05) when compared with those obtained after 48 hr of treatment (Figures 3A and 3B, respectively). We focused only on the miRNAs significantly induced at both time points and with higher expression levels: *miR-132*, *miR-21*, and *miR-212* (Figure 3C). Interestingly, the three miRNAs show high *in silico* binding affinity on the *COUP-TFI* 3′UTR, as predicted by the miRanda bioinformatics tool (Betel et al., 2008) (Figure 3D, Data S1).

As the three miRNAs represent good candidates for the embryonic regulation of COUP-TFI expression during corticogenesis, we directly tested their function by inhibiting their action. *miR-132* and *miR-212* are organized in a transcriptional cluster and share the same seed sequence and putative targets (Wanet et al., 2012). To inhibit the function of both *miR-21* and *miR-132/miR-212,* we targeted their seed sequences by transfecting locked nucleic acid (LNA)-based antisense oligonucleotides in corticalized mESCs (Stenvang et al., 2012). We found that on inactivating both *miR-21* and *miR-132/miR-212 in vitro*, the ratio of cells expressing COUP-TFI in cultures treated with FGF8 significantly increases compared with scramble LNA control transfections (Figures 4A–4C). This supports the involvement of miRNAs in mediating FGF8-dependent inhibition of COUP-TFI *in vitro*.

However, the induction of the three miRNAs by FGF8 *in vitro* might not reflect the actual *in vivo* mechanisms of COUP-TFI regulation. To this purpose, we investigated whether they were differentially expressed in anterior and posterior regions of the embryonic mouse cortex at E11.5, a developmental time in which the A/P COUP-TFI expression gradient is well defined (Armentano et al., 2007). If these miRNAs were involved in COUP-TFI translational inhibition, we would expect higher expression in anterior cortical regions where COUP-TFI expression is normally low. Among the three miRNAs, only *miR-21* is significantly ($p < 0.05$) more expressed in the anterior than posterior cortex, whereas *miR-132* and *miR-212* show no regionalized expression (Figure 4D). Moreover, *miR-21* expression is significantly increased in E11.5 *COUP-TFI null* cortices, which have expanded rostral features (Armentano et al., 2007; Alfano et al., 2014), whereas *miR-132* and two unrelated control cortical miRNAs (*miR-181* and *miR-222*) are not affected (Figure 4E), indicating that *miR-21* is linked to anterior cortical identity.

Since *miR-21* shows the most promising expression pattern and *miR-132* has two predicted binding sites with high affinity, we focused on these two miRNAs and investigated whether they could interfere with the expression of *COUP-TFI*, *Sp8*, or *Fgf8*. Transfection of mature *miR-21* in DIV8 cells affects only the *COUP-TFI* transcript level at DIV10, leaving *Sp8* and *Fgf8* mRNA expression unchanged,

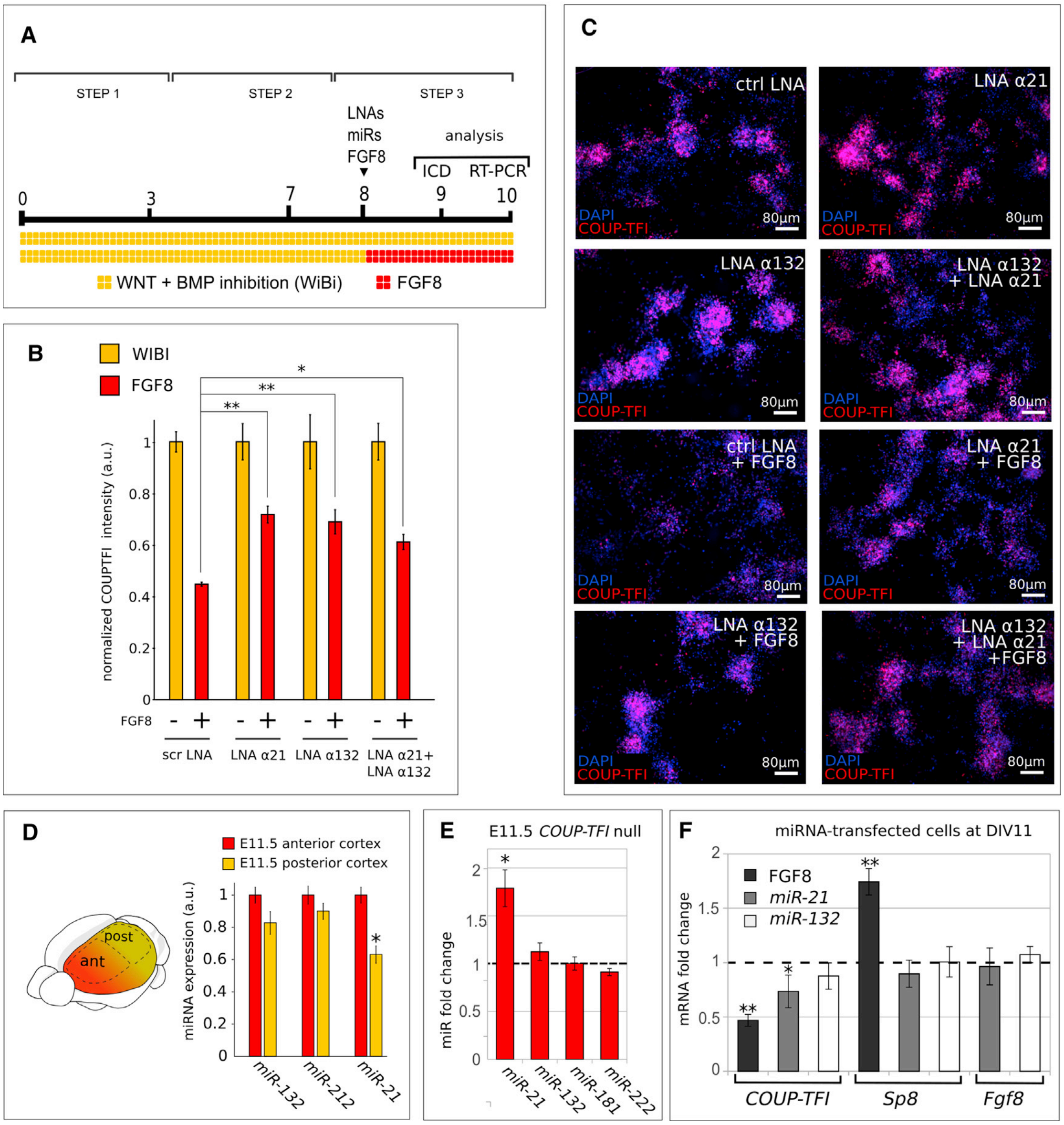

Figure 4. FGF8-Induced miRNAs Repress COUP-TFI Expression *In Vitro*

(A) Schematics of the cell sample preparation for the LNA experiment.

(B) Normalized COUP-TFI fluorescence intensity (see Experimental Procedures) in cells transfected with LNA against *miR-21*, *miR-132/212* (LNA α21 and LNA α132, respectively) or scramble LNA (ctrl LNA) and treated with FGF8 for 24 hr (n = 3 independent cultures). Error bars correspond to SEM. Statistical significance was obtained via the one-way ANOVA ($p < 0.005$), followed by the t test with Bonferroni correction ($^*p \leq 0.05$, $^{**}p \leq 0.01$).

(C) Immunocytodetection of COUP-TFI (red) in cells transfected with LNA against different *miRNAs* (α, in labels) and treated with FGF8 for 24 hr.

(D) Normalized levels of *miR-21*, *miR-132*, and *miR-212* in anterior or posterior E11.5 mouse embryonic cortex dissected as shown in the left cartoon and analyzed by RT-PCR. Error bars represent SEM (n = 3 independent experiments); $^*p \leq 0.05$, Student's t test.

(E) RT-PCR analysis showing fold changes in miRNA expression in E11.5 *COUP-TFI null* normalized to WT cortices. Error bars represent SEM (n = 3 independent cultures); $^*p \leq 0.05$, Student's t test.

(F) RT-PCR mRNA analysis of DIV11 progenitors *in vitro* after transduction with miRNAs or treatment with FGF8. Error bars represent SEM (n = 3 independent cultures); $^*p \leq 0.05$, $^{**}p \leq 0.01$, Student's t test.

whereas *miR-132* overexpression has no effect on the expression of the three genes (Figure 4F). Finally, the analysis of Ki67-positive dividing progenitors at DIV11 after the transfection of mature *miR-21* at DIV8 showed no effect on neural progenitor proliferation *in vitro* (Figure S2), in line with unchanged *Sp8* and *Fgf8* expression. Taken together, our data suggest that *miR-21* might be the most relevant miRNA for establishing COUP-TFI graded expression and that this effect would be exerted downstream of FGF8.

Specificity of *miR-21* Interaction with the *COUP-TFI* 3′UTR

To further decipher the functional specificity of *miR-21* and *miR-132* on the *COUP-TFI* 3′UTR, COUP-TFI protein levels were assessed after mature miRNA lipofection at DIV8. *miR-21* transfected cells have lower protein levels than cells lipofected with *miR-132* or controls (Figures 5A–5E), in accordance with the mRNA analysis by RT-PCR (Figure 4F). Next, we investigated *miR-21* and *miR-132* specificity by mutating the seed sequence of their predicted binding sites in *COUP-TFI* 3′UTR (Figure S3) and compared the effects of different mutations *via* EGFP reporter assay in transfected HEK293T cells. Both *miR-21* and *miR-132* significantly inhibit EGFP reporter translation in the presence of wild-type (WT) seed but are ineffective when seeds are mutated, thus confirming their specificity of action (Figure 5F).

Finally, we assayed the EGFP WT 3′UTR vector in DIV8 corticalized cells and observed EGFP signal inhibition 48 hr after FGF8 treatment (Figures 5G–5K), thus suggesting that the effect of FGF8 on COUP-TFI translation is most likely direct and mediated by signals in the 3′UTR. Moreover, transduction of both *miR-21* and *miR-132* mutated vectors in FGF8-treated cells shows intermediate EGFP levels, but only the mutation of the *miR-21* binding site significantly decreases the inhibitory effect of FGF8 compared with WT (Figure 5K). Taken together, these data confirm a preferential role of *miR-21* in mediating the inhibitory effect of FGF8 on COUP-TFI in corticalized cells.

Complementary Expression of *miR-21* and COUP-TFI Protein and their Interaction *In Vivo*

To evaluate the *in vivo* role of *miR-21* on COUP-TFI regulation, we compared *miR-21* and COUP-TFI expression in E12.5 mouse embryonic cortices. Mature *miR-21* shows decreasing A/P and M/L expression gradients (Figures 6A–6D), which are complementary to the increasing A/P and M/L COUP-TFI protein gradient (Figures 6E–6H), and in line with the RT-PCR-based expression analysis (Figure 4D). This suggests that *miR-21* could regulate COUP-TFI expression also *in vivo* by interacting with its 3′UTR.

To directly test this hypothesis, we first overexpressed a construct encompassing the *COUP-TFI* 3′UTR linked to a GFP reporter (Figure 7A) and then inactivated *miR-21* action by *in vivo* lipofection using specific LNA oligonucleotides (Figure 7B). *In utero* electroporation (IUE) of the *COUP-TFI* 3′UTR plasmid into E12.5 embryos (Figure 7A) increases COUP-TFI levels in GFP-electroporated progenitors after 48 hr (Figures 7D, 7F, and 7G), compared with the contralateral non-electroporated cortex or to control GFP-electroporated brains (Figures 7C, 7E, and 7G). IUE of the *COUP-TFI* 3′UTR plasmid carrying a mutated *miR-21* binding site does not affect the COUP-TFI protein levels (Figure S4). Thus, similarly to the *in vitro* data (Figure 2), abundant levels of the exogenous *COUP-TFI* 3′UTR might act as a "sponge," competing with its endogenous counterpart by sequestering available miRNA and hence counteracting COUP-TFI inhibition.

To further support *miR-21* action in COUP-TFI inhibition, we lipofected E12.5 cortices with LNA-based antisense oligonucleotides against *miR-21* (Figure 7B) and tested COUP-TFI protein levels. Notably, we found increased levels in the lipofected E14.5 anterior embryonic cortex (Figures 7I, 7K, and 7M) compared with the contralateral non-lipofected hemisphere (Figures 7H, 7J, and 7M), or to the side lipofected with a scramble non-specific LNA (Figures 7L and S5A–S5D), indicating that blocking *miR-21* action affects COUP-TFI translation *in vivo*. No significant differences in COUP-TFI levels were found between treated and non-treated E12.5 medial and posterior cortices (Figure 7M), consistent with a higher concentration of *miR-21* in anterior versus posterior cortex (Figures 6A–6D). Together, these data confirm a key role of *miR-21* in inhibiting COUP-TFI levels *in vivo*.

Overall, our *in vivo* approaches support the *in vitro* data and strongly suggest that *miR-21* is a key component in mediating the FGF8 action of COUP-TFI anterior inhibition by cooperating in the establishment of its low anterior to high posterior expression gradient observed in embryonic cortices.

DISCUSSION

We found that corticalized mESCs express significant levels of COUP-TFI and that FGF8 treatment can inhibit COUP-TFI during a precise time of the neuralization protocol. This reproduces well what normally happens during early corticogenesis, in which FGF8 downregulates anterior COUP-TFI expression in progenitor cells (reviewed in Alfano and Studer, 2013). We showed that the mechanisms of COUP-TFI inhibition exerted by FGF8 *in vivo* can be reproduced *in vitro* and that FGF8 can initiate a genuine program of anterior positional identity specification in a cell culture system. Our experiments not only confirmed the reliability of our *in vitro* approach but also revealed an unexpected mechanism of post-transcriptional regulation,

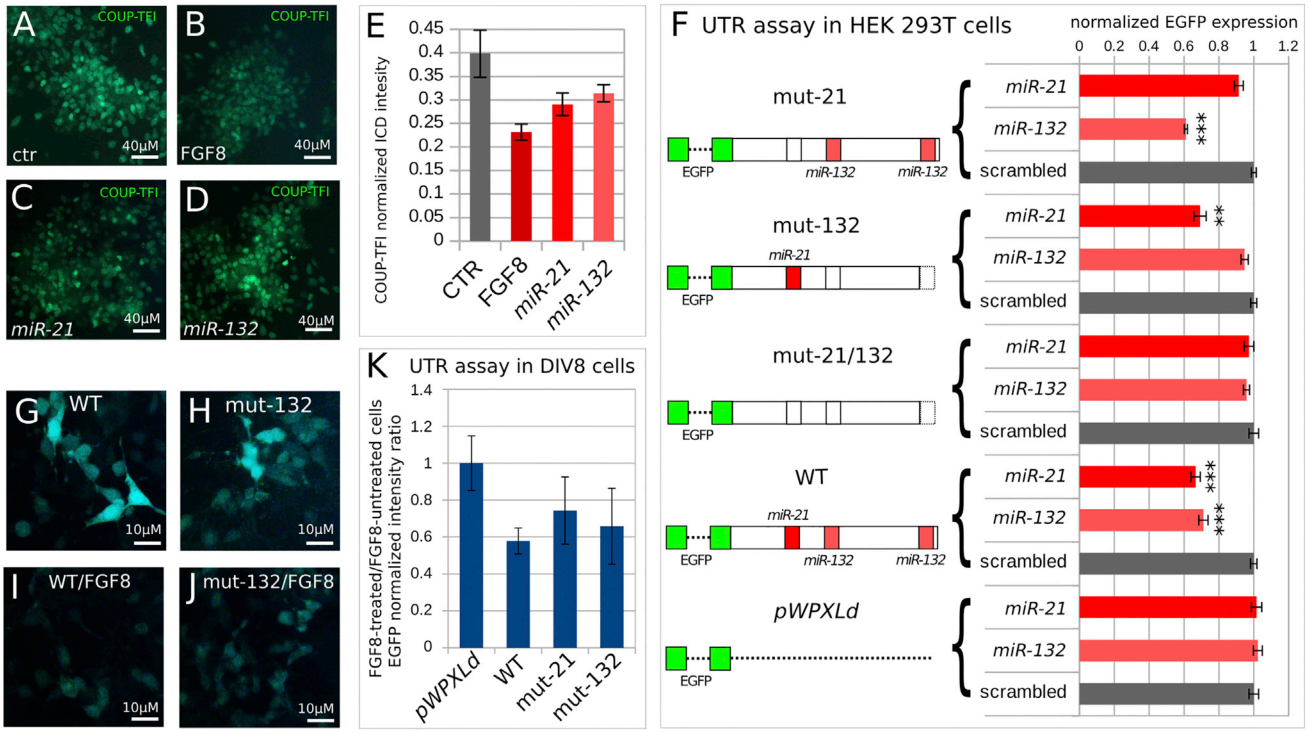

Figure 5. *mir-21* and *miR-132* Selective Binding to the *COUP-TFI* 3′UTR

(A–D) Examples of cells transfected with control (ctr) miRNA, *miR-21*, or *miR-132* at DIV8, cultured to DIV11 in normal or FGF8-containing medium, as indicated in the images, and then immunostained with COUP-TFI antibody at DIV11.

(E) Quantification of COUP-TFI protein levels of transduced cells in (A–D). Relative intensity of COUP-TFI antibody fluorescence was normalized with respect to DAPI signal; n = 2 independent cultures.

(F) Effect of the mutation of miRNA seed on the translation of reporter constructs carrying EGFP after their transfection in HEK293T cells and EGFP imaging 24 hr after transfection. Fluorescence intensity values were normalized with respect to cells transfected with control miRNA containing a scrambled sequence.

(G–J) DIV10 cells after lentiviral transduction of EGFP reporter constructs as in (F) and 48 hr of culture in normal medium (G and H) or in medium containing FGF8 (I and J).

(K) Effect of the mutation of miRNA seed on the translation of reporter constructs carrying EGFP after their transduction in DIV8 cells and EGFP imaging 48 hr after transduction. Values show the ratio of fluorescence between cells cultured without FGF8 and cells treated with FGF8 from DIV8 to DIV10, after normalization to the ratio of control cells transduced with pWPXLd.

EGFP intensity evaluation shown in (F) and (K) was calculated as EGFP/DAPI pixel intensity ratio in cells from n = 3 (F) or n = 2 (K) independent cultures. In (F), $^{**}p \leq 0.01$ and $^{***}p \leq 0.001$, Student's t test.

acting through the 3′UTR of *COUP-TFI* also *in vivo*. FGF8 signaling initially decreases COUP-TFI protein levels without affecting *COUP-TFI* mRNA, and this effect is blocked by overexpressing its 3′UTR, which likely acts as a scavenging sponge for FGF-induced miRNAs. Our data demonstrate that *miR-21* represents one of the key mediators in the FGF8-dependent COUP-TFI inhibition *in vitro* and *in vivo*. Overall, we propose that cell culture systems can mimic the differentiation of specific neuronal types and be used in unraveling molecular mechanisms of cortical positional patterning.

Despite resembling their physiological counterparts, simple cell culture systems lack the endogenous morphogenic gradients normally present *in vivo*, such as FGF8. However, this can be an advantage when performing functional experiments aiming to identify the mechanisms involved in the formation of graded responses to a known signal. Indeed, by comparing the miRNAome of early cortical progenitor cells in the presence or absence of FGF8, we found few miRNAs immediately induced by FGF8. Only three miRNAs predicted to target the *COUP-TFI* 3′UTR were significantly induced by 24 hr of FGF8 treatment (*miR-21*, *miR-132*, and *miR-212*). Consistently, FGF signaling was reported to induce *miR-132* expression in endothelial cells (Anand et al., 2010), indicating that multiple tissues share a molecular mechanism of *miR-132*

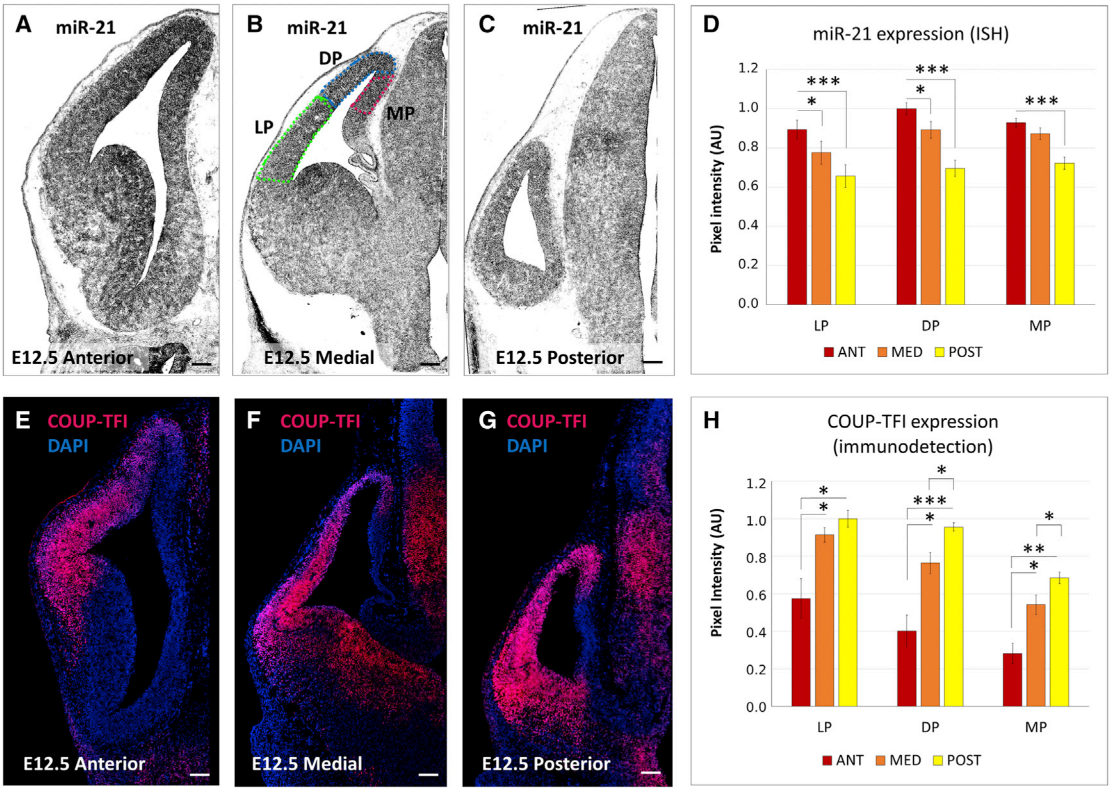

Figure 6. ***miR-21*** **and COUP-TFI Are Expressed in Complementary Gradients in the Embryonic Cortex**

(A–C) *In situ* hybridization of *miR-21* in E12.5 mouse brain sections at anterior (A), medial (B), and posterior (C) levels along the A/P brain axis. Dotted outlines in (B) indicate the areas (LP, lateral pallium, DP, dorsal pallium, MP, medial pallium) where signal intensity was measured. Scale bars, 100 μm.

(D) Normalized COUP-TFI intensity (ImageJ, pixel intensity analysis) in different pallial regions as schematized in (B). Measurements were performed on at least three sections from the anterior, medial, and posterior regions of E12.5 brains (n = 3), in different dorsoventral areas as indicated (LP, lateral pallium; DP, dorsal pallium; MP, medial pallium). Error bars, SEM. Statistical significance obtained by Student's t test (*p ≤ 0.05, ***p ≤ 0.001).

(E–G) Immunofluorescence of COUP-TFI (red) on E12.5 brain sections at different A/P levels as in (A)–(C). Nuclei counterstaining (blue) was obtained with DAPI. Scale bars, 100 μm.

(H) Normalized COUP-TFI fluorescence intensity (ImageJ, pixel intensity analysis) in different pallial regions of E12.5 brains (n = 3), as indicated. Measurements were performed as in (D). Error bars, SEM. Significance obtained by Student's t test (*p ≤ 0.05, **p ≤ 0.01, ***p ≤ 0.001).

activation by FGF. In our study, all three miRNAs were able to inhibit COUP-TFI translation in corticalized ESCs, but only *miR-21* is expressed in a gradient complementary to COUP-TFI in embryonic cortices. This does not imply that *miR132* and *miR-212* have no effect on COUP-TFI expression, but that, possibly, *miR-21* is the most effective one in modulating cortical COUP-TFI gradient expression, whereas *miR-132* and *miR-212* might fine-tune COUP-TFI in other contexts and/or temporal windows.

As COUP-TF members are orphan nuclear receptors, for which no ligands have been characterized so far, it is plausible that their expression depends on regulatory feedforward and feedback loops involving miRNAs in several developmental and differentiation processes (Eendebak et al., 2011). Indeed, several papers unraveled a co-regulation between COUP-TFII (also Nr2f2) and *miR-302* in ESC differentiation (Rosa and Brivanlou, 2011; Hu et al., 2013; Wang et al., 2014; Kang et al., 2015), or COUP-TFII and other miRNAs in various forms of cancer (Lichtfield and Klinge, 2012; Qin et al., 2014). However, very little was known about the regulation of COUP-TFI expression by miRNAs during neural development. One paper predicted a series of miRNAs that could target COUP-TFI during inner ear development and uncovered a co-regulatory interaction between COUP-TFI and *miR-140* on KLF9 expression (Chiang et al., 2013). This interaction seems not to work

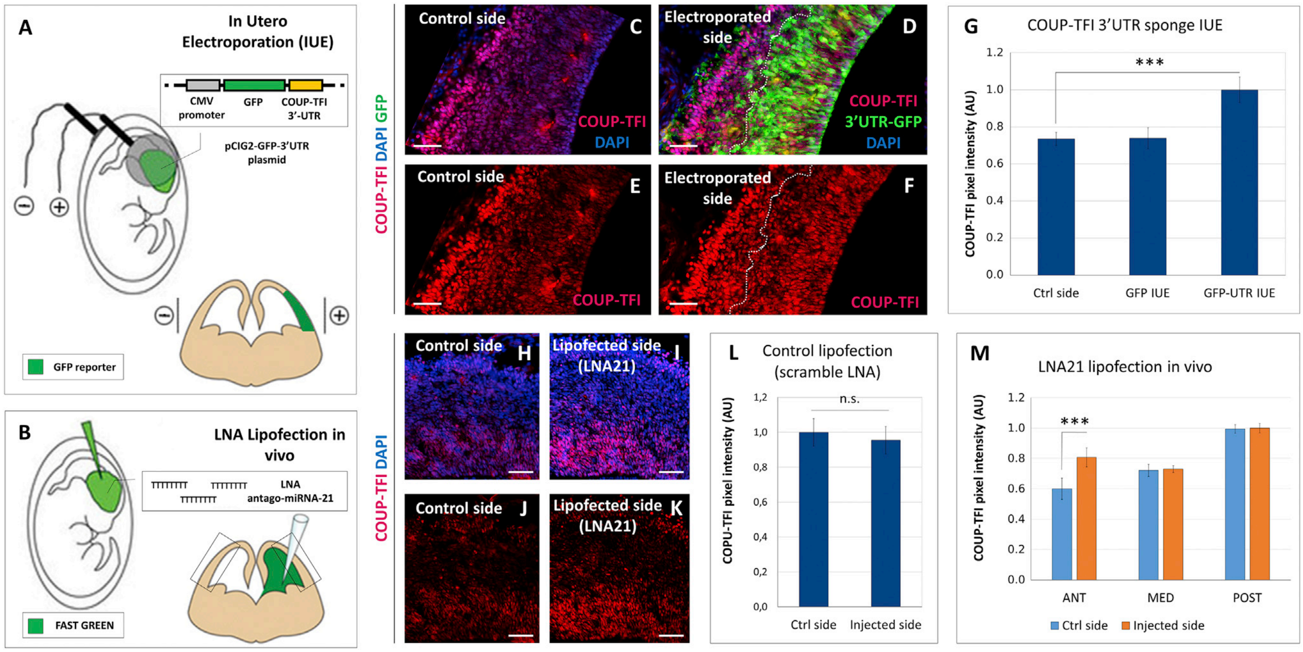

Figure 7. *miR-21*-Mediated Control of COUP-TFI Translation *In Vivo*

(A) Schematics of the *in utero* electroporation (IUE) experiment. The *pCIG2-IRES-GFP-3′UTR* "sponge" plasmid was injected into one telencephalic hemisphere and electroporated in neural progenitors, as described in Experimental Procedures. To target the lateral/dorsal pallium of one hemisphere, the electrodes were placed as schematized. Brains were collected 48 hr later and processed for IF. The electroporated area was recognizable by GFP expression.

(B) Schematics of the LNA experiment *in utero*. Brains were injected with Lipofectamine/LNA mixture at E12.5 and collected 48 hr later for IF. The injected side (visualized by adding fast green to the LNA mixture) was compared to the contralateral un-injected side.

(C–F) IF of COUP-TFI (red) and GFP (green) in brain sections electroporated with the "sponge" plasmid at E12.5 and analyzed at E14.5. The lateral/dorsal pallium of the electroporated hemisphere (D and F) was compared with the contralateral control hemisphere (C and E). Nuclei counterstaining (blue) was obtained with DAPI. Dotted outlines in (D) and (F) indicate the GFP-positive area where COUP-TFI fluorescence signal intensity was quantified. Scale bars, 50 μm.

(G) Pixel intensity quantification (ImageJ) of COUP-TFI fluorescence in electroporated or control cortices (n = 3 brains per group). The staining was quantified only in the ventricular (VZ) and subventricular zones (SVZ), where cortical progenitors reside, as electroporated cells need more than 2 days to reach the cortical plate. Error bars, SEM; t test (***$p \leq 0.001$).

(H–K) IF of COUP-TFI (red) in brain sections after *miR-21* inhibition by means of LNA lipofection *in utero*. The neural progenitor regions of the lipofected hemisphere (I and K) were compared with equivalent regions of the contralateral control hemisphere (H and J). Nuclei counterstaining (blue) was obtained with DAPI. Scale bars, 50 μm.

(L) Pixel intensity quantification (ImageJ) of COUP-TFI fluorescence in cortices lipofected with control scramble LNA, compared with non-lipofected cortex (n = 3 brains). Corresponding images are shown in Figures S5A–S5D. Error bars, SEM; t test (n.s., non-significant).

(M) Pixel intensity quantification (ImageJ) of COUP-TFI fluorescence in cortical areas (n = 3 brains) as shown in (H)–(K). The staining intensity was quantified at different levels along the A/P axis of E14.5 brains, as indicated. A significant difference was found only in the injected side of anterior regions. Error bars, SEM; t test (***$p \leq 0.001$).

in the cerebral cortex, indicating that miRNAs might regulate COUP-TFI expression in a cellular-dependent context. Finally, *miR-17/106* might act as critical regulators of the neurogenic-to-gliogenic transition in which both *COUP-TF* genes are also involved, however no direct regulation between COUP-TFs and *miR-17/106* has been described (Naka-Kaneda et al., 2014). On the contrary, our results reveal a specific role of *miR-21* in regulating COUP-TFI protein expression in early cortical progenitor cells. Therefore, this is the only study, to our knowledge, demonstrating fine regulation of COUP-TFI expression by a miRNA, *miR-21*, during neocortical area patterning. Finally, it would be interesting to find out whether either *miR-21* or other *miRNAs* regulate or are controlled by other transcription factors involved in cortical area specification.

In conclusion, our observations indicate that molecular mechanisms regulating genes of cortical patterning are maintained in isolated cells *in vitro*, allowing the use of cell culture models for studies of cortical areal development. At the same time, they open new opportunities for

therapeutic approaches in which the differentiation of pluripotent cells into a motor rather than a sensory cortical neuron is required for cell transplantation experiments in a damaged/degenerated cerebral cortex.

EXPERIMENTAL PROCEDURES

All mouse experiments were conducted according to national and international guidelines and have been approved by the local ethical committee (CIEPAL NCE/2014-209).

Mouse ESC Differentiation and Transfection

Murine ESC lines E14Tg2A (passages 25–38) were cultured on gelatin-coated tissue culture dishes at a density of 40,000 cells/cm^2. ESC medium, changed daily, contained GMEM (Sigma), 10% fetal calf serum, 2 mM glutamine, 1 mM sodium pyruvate, 1 mM non-essential amino acids (NEAA), 0.05 mM β-mercaptoethanol, 100 U/mL penicillin/streptomycin, and 1,000 U/mL recombinant mouse leukemia inhibitory factor (Invitrogen). Chemically defined minimal medium (CDMM) for neural induction consisted of DMEM/F12 (Invitrogen), 2 mM glutamine, 1 mM sodium pyruvate, 0.1 mM NEAA, 0.05 mM β-mercaptoethanol, 100 U/mL penicillin/streptomycin supplemented with N2/B27 (no vitamin A; Invitrogen). The differentiation protocol is organized in three steps. In step I, ESCs were dissociated, washed with DMEM/F12, and seeded on gelatin-coated culture dishes (65,000 cells per cm^2), then cultured in CDMM plus 2.5 μM WNT inhibitor (53AH, Cellagen Technology) and 0.25 μM BMP inhibitor (LDN193189, Sigma) for 3 days. In step II, ESCs were dissociated and seeded (65,000 cells per cm^2) on poly-ornithine (Sigma; 20 μg/mL in sterile water, 24 hr coating at 37°C) and natural mouse laminin (Invitrogen; 2.5 μg/mL in PBS, 24 hr coating at 37°C). Cells were maintained for 4 additional days in CDMM plus WNT/BMP inhibitors, changing the medium daily. Several washes with DMEM/F12 were used to remove the serum employed for trypsin inactivation. In step III, cells were dissociated and seeded (125,000 cells per cm^2) on poly-ornithine- and laminin-coated wells. Subsequently, WIBI cultures were kept in CDMM plus WNT/BMP inhibitors for 4 (WiBi 11) additional days. FGF8-treated cells (FGF8) were grown in CDMM supplemented with FGF 100 ng/mL (R&D), replaced daily. MEK inhibitor (PD0325901, Calbiochem, 1 μM; Barrett et al., 2008) was added daily to block FGF signaling. On the 11th day of differentiation *in vitro*, DMEM/F12 was replaced with Neurobasal medium, and NEAA were removed from the CDMM to avoid glutamate-induced excitotoxicity.

LNA miRNA transfection was performed using Lipofectamine 2000 (Invitrogen) according to the manufacturer's instructions (miRCURY LNA miRNA Inhibitors, Exiqon). Briefly, each LNA (5 nmol) was resuspended in TE buffer (10 mM Tris pH 7.5 or 8.0, 1 mM EDTA) to a concentration of 50 μM. Cells were then transfected overnight at 37°C and 5% CO_2 in 24-well plates using 80 nM LNA and 2.5 μL/well of Lipofectamine 2000 in a final volume of 0.5 mL/well Opti-MEM. Since an LNA concentration higher than 100 nM was toxic for the cells, we used less than half the concentration (40 nM) in the experiments in which two LNAs were co-transfected. Mature miRNAs (miRNA mimics, supplied by Shanghai GenePharma) were transfected in 24-well plates at 60 nM final concentration using 2 μL of Lipofectamine 2000 in 0.5 mL of Opti-MEM for 4–6 hr. mmu-miR-21a-5p mimic was assembled by annealing uagcuuaucagacugauguuga-uu sense strand RNA and ucaacaucagucugauaagaaa-uu antisense strand RNA for 10 s at room temperature. For mmu-miR-132-3p mimic, uaacagucuacagccauggucg-uu sense strand and cgaccauggcuguaga cugaaa-uu antisense strand RNAs were used. Scramble control miRNA had uucuccgaacgugucacgu sense strand and acgugacac guucggagaa antisense strand. When co-transfected with lentiviral vectors carrying WT or mutated *COUP-TFI* 3′UTR in HEK293T cells, miRNA mimics were used at 30 nM final concentration and mixed with 500 ng of lentiviral vector DNA.

Gene Expression Analyses

RT-PCR and immunocytodetection (ICD) on cultured cells were carried out as previously described (Bertacchi et al., 2013). A more detailed description of these methods is reported in Supplemental Information.

For western blot analysis, sample lysis was performed using RIPA buffer (50 mM Tris-HCl [pH 7.6], 1% NP-40, 0.5% deoxycholic acid, 150 mM NaCl, 1 mM EDTA, 1 mM PMSF, 1% SDS) supplemented with complete protease inhibitor cocktail (Roche). Lysates were then incubated for 30 min on ice and sonicated three times for 10 s each on medium power in order to reduce viscosity. The supernatant was harvested by centrifugation (10 min at 13,000 rpm, 4°C) and quantified with a Micro BCA Protein Assay Kit (Thermo Scientific). Samples were then denatured with LDS sample buffer (Thermo Scientific) and heated for 10 min on a thermal block at 99°C. The total protein extract (10–20 μg) was resolved on 10% acrylamide gels, transferred onto a nitrocellulose membrane (Hybond-c Extra, GE Healthcare), blocked with 5% milk proteins in TBST (50 mM Tris [pH 7.6], 150 mM NaCl, 0.05% Tween 20), and incubated for 1 hr at room temperature with primary antibodies: COUP-TFI (1:2,000; Abcam ab60059) and α-tubulin (Sigma-Aldrich T6074, 1:5,000). The membrane was washed three times with TBST (15 min each) and probed with an horseradish-peroxidase-conjugated secondary anti-mouse or anti-rabbit antibody for 1 hr (Santa Cruz Biotechnology; sc-2005 and sc-2030, respectively). After three more washes, the signal was revealed by means of an enhanced chemiluminescence kit (G&E Healthcare) on a BioMax XAR Film (Kodak). Densitometric analysis of the blots was performed with either ImageJ (imagej.nih.gov) gel analysis function or the ChemiDoc Imaging Systems Software (Bio-Rad).

For immunodetection on cortical section, mouse embryonic brains were dissected and fixed in 4% paraformaldehyde (PFA) at 4°C for 2 hr in agitation, then washed in PBS 1× and dehydrated in 30% sucrose overnight at 4°C. Brains were then embedded in optimal cutting temperature compound (OCT) and stored at −80°C. Cryostat tissue sections (14 μm) were processed for immunodetection, as described for *in vitro* ICD, with the only difference that antigen-retrieval unmasking was performed at the beginning of the staining by boiling slides twice in sodium citrate 85 mM (pH 6).

For RNA *in situ* hybridization (ISH), *miRNA-21* antisense and control scramble probes (Exiqon) were used following the manufacturer's instruction. Brains for ISH were fixed overnight with PFA 4% at 4°C, dehydrated in 30% sucrose, and embedded in OCT

resin, then stored at −80°C. ISH was carried out on 14-μm cryosections as follows. Defrosted and air-dried brain sections were treated with RIPA buffer (150 nM NaCl, 1% NP-40, 0.5% Na deoxycholate, 0.1% SDS, 1 mM EDTA, 50 mM Tris [pH 8.0]) for 10 min, then post-fixed for 15 min in 4% PFA at room temperature. Pre-hybridization and hybridization of the sections with Exiqon probes were performed in the following solution: 50% formamide, 5× saline sodium citrate (SSC), 5× Denhardt's solution (Invitrogen), 500 μg/mL salmon sperm DNA (Ambion), 250 μg/mL yeast tRNA. The Exiqon probe was added to the hybridization solution at a concentration of 0.5 μL for 300 μL/slide. Different temperatures were tested for optimal hybridization, ranging from 4°C to 37°C; best results were obtained with cold temperature (4°C). The day after, slides were washed twice for 1 hr at 4°C in the following solution: 50% formamide, 2× SSC, 0.1% Tween 20. Then, the samples were equilibrated in MABT (maleic acid buffer containing Tween 20) solution and blocked in MABT/10% sheep serum. The hybridized probes were detected by overnight incubation at 4°C with an anti-DIG antibody (ENZO ENZ-ABS266-0100, 1:2,000). Finally, sections were washed several times in MABT, then equilibrated in B3 buffer (100 mM Tris [pH 9.5], 50 mM $MgCl_2$, 100 mM NaCl, 0.1% Tween 20). The staining was performed by incubating the slices in NBT/BCIP solution (nitro-blue tetrazolium chloride and 5-bromo-4-chloro-3′-indolyphosphate p-toluidine salt; Roche) at room temperature (or overnight at 4°C). Two quick washes with B3 buffer supplemented with tetramisole (0.5 mg/mL; Sigma) were performed before staining to reduce background.

Cell Imaging

Immunofluorescence microphotographs were processed and analyzed with ImageJ 1.48p software (https://imagej.nih.gov/ij/index.html), measuring integrated pixel intensity. COUP-TFI signal quantification was performed on three biological replicas per group and on ten different randomly chosen fields for each sample. Analysis of COUP-TFI expression was performed by first applying an appropriate threshold for each channel in order to remove background signal, then normalizing COUP-TFI pixel intensity on the nuclear staining (DAPI). EGFP reporter activity in HEK293T and neuralized ESCs was evaluated as EGFP/DAPI pixel intensity ratio. For HEK293T analysis, three independent cultures were imaged (n = 9 random fields per treatment). For neuralized ESCs, ten different randomly chosen fields of two biological replicas per group were analyzed.

Lentiviral Vector Construction and Use

The entire COUP-TFI 3′UTR (748 bp) was obtained from the genomic BAC library RPCI-23 (Source Bioscience, Genome Cube) and amplified via PCR using a forward and reverse primer carrying, respectively, an XmaI and KpnI restriction site at their 5′ (forward, attcccgggactttgggtgtttcccaccc; reverse, taaaggtaccttttgctaaattctttta tttttgtttaa). The vector carrying the *COUP-TFI* 3′UTR (virCOUP) was constructed swapping the original WPRE sequence in the pWPXLd vector (Addgene number 12,258), with the amplicon carrying the *COUP-TFI* 3′UTR using the restriction sites XmaI/KpnI. The ligated vector was then sequenced to ensure the correct cloning of the 3′UTR.

Mutations of the predicted binding sites of mmu-miR-21a-5p (miR-21) and mmu-miR-132-3p (miR-132) in the 3′ UTR of *COUP-TFI* were obtained by PCR using Q5 High-Fidelity DNA Polymerase (NEB). The seed sequence of miR-21 at +245 and that of miR-132 at +389 (Figure S3) were replaced with a NotI restriction sequence. To this aim, upstream and downstream halves of mutated 3′UTR were generated by PCR using the external forward or reverse primer reported above in combination with a mutated internal reverse or forward primer, respectively. The mutated internal primers used for miR-21 site were miR-21_mut_fw caccaagcggccgcgatttggaagagaggaccatgag and mir-21_mut_rev cacaggcggccgcacgaggtcctttttctttcctttccaatgtac. The mutated internal primers used for miR-132 site at +389 were miR-132_mut_fw cagtatgcggccgcaatcctatgtagaaacatacactgaacattgt tattc and mir-132_mut_rev cagctagcggccgcttccatatgagtagttttctgta cagaatatatcc. Upstream and downstream mutated halves were then digested with NotI enzyme, ligated, and used as a template for PCR together with external forward and reverse primers. PCR products were finally restricted with XmaI/KpnI, purified, and inserted in pWPXLd vector. The seed sequence of miR-132 at +651 was deleted by PCR. For this purpose, the 3′UTR with the mutated miR-132 site at +389 was used as a template, and forward attcccgggactttgggtgtttcccaccc and reverse taaaggtacccgacaacata tatcgcactcattataagaagc as primers. The deleted fragment obtained was then restricted with XmaI/KpnI, purified, and cloned in pWPXLd vector.

Lentiviral vectors were produced transfecting 293T cells with 150 nM polyethyleneimine reagent (Sigma) and either pWPXLd-memGFP or pWPXLd-memCherry plasmids, together with the Δ8.91 packaging and a VSV-G envelope expressing plasmids (Zufferey et al., 1997) in a ratio of 20 μg:15 μg:5 μg per single 100-mm dish. Transfection medium was discarded 24 hr after transfection, and viral particles were collected 48 hr and 72 hr after transfection, pooled, and frozen at −80°C.

miRNAome Profiling

For each point, two biological replicates, each consisting of a pool of three cell cultures, were analyzed. Total RNA was extracted with miRNeasy Mini Kit (QIAGEN). The small-RNA library was prepared using 1 μg of total RNA per sample and the TruSeq Small RNA Sample Preparation Kit (Illumina) following the manufacturer's instructions and sequenced on an Illumina HiSeq 2000 platform, running in 50-bp single-read mode using sequencing chemistry v3, and then demultiplexed in an FASTQ format using CASAVA v.1.8 (Illumina). Library adaptors were trimmed and reads were mapped to the mouse genome (NCBI37/mm9) with miRExpress (Wang et al., 2009). miRNA reads were annotated using the miRBase mouse reference (mirbase.org, V19). Normalization was performed as counts per million and differential expression was evaluated with the R package EdgeR (Robinson et al., 2010; Bioconductor).

In Silico Analysis of 3′UTR Putative Binding Sites

For the identification of potential binding sites in the COUP-TFI 3′UTR, we employed the miRanda algorithm (Betel et al., 2008; Enright et al., 2003; microrna.org), which takes into account both the seed complementarity score and the thermodynamic stability of the binding between miRNAs and 3′UTRs (Data S1, Figure 3D),

setting as thresholds a minimum score of 100 and a maximum energy of −15 kcal/mol.

In Utero Electroporation

In utero electroporation was performed on E12.5 mouse brains, by targeting the latero-dorsal regions of the telencephalic dorsal pallium. The electroporations were performed using a Tweezertrode electrode (diameter 7 mm; BTX) connected to a NEPA21 Type-II electroporator (NEPA GENE). The following parameters were used: four 35 V pulses, P(on) 50 ms, P(off) 1 s and 5% decay rate. The "sponge" plasmid was produced by cloning the PCR-amplified COUP-TFI 3′UTR DNA sequence into the XhoI and NotI sites of a *pCIG2-IRES-GFP* plasmid (Heng et al., 2008). The "mutated sponge" (where miR-21 seed was substituted by a scramble sequence, see Figure S4A) was obtained by inserting the mutation with apposite primers (5′-caacttgcttaaaatgaaaaaaaaaaaaaaaaaaaaagaac gatttg-3′; 5′-ttttcattttaagcaagttgacgaggtcctttttctttcctttcc-3′) then amplifying the mutated UTR sequence with NotI site containing primers (5′-ataagaatgcggccgcgactttgggtgtttcccacccaat-3′; 5′-ataa gaatgcggccgcttttgctaaattcttttattttg-3′) and finally cloning it in the pCIG2-IRES-GFP plasmid. The 1× DNA solution for IUE was injected in one of the two brain telencephalic vesicles prior to the application of electric current and consisted of endo-free TE buffer, 1× fast green, and the plasmid at a final concentration of 1 mg/mL. The original *pCIG2-IRES-GFP* plasmid was electroporated at the same concentration as the control. For LNA lipofection experiments, the injected solution consisted of 3 μL of Lipofectamine 2000 (Invitrogen), 0.4 μL of fast green, and 2 μL of LNA antagomir. In this case, since the transfection was mediated by Lipofectamine, no electric field was applied following the injection into telencephalic vesicles.

ACCESSION NUMBERS

The accession number for the microRNA sequencing data reported in this paper is GEO: GSE117932.

SUPPLEMENTAL INFORMATION

Supplemental Information includes Supplemental Experimental Procedures, five figures, one table, and one data file and can be found with this article online at https://doi.org/10.1016/j.stemcr.2018.08.002.

AUTHOR CONTRIBUTIONS

M.T., M. Bertacchi., and F.C. designed experiments; M.T. performed cell culture, molecular biology, imaging, and gene expression data computation; M.S. and M. Bertacchi. planned and carried out *in vivo* experimental activity and imaging; M.C. performed molecular cloning; M. Baumgart designed and performed the microRNA sequencing; A.C. performed the microRNA sequencing computational analysis; M.S. and F.C. wrote the manuscript.

ACKNOWLEDGMENTS

We thank Dr. Marco Groth from the core facility DNA sequencing of the Leibniz Institute on Aging - Fritz Lipmann Institute in Jena for his help with miRNA sequencing and Greta Ghiloni for technical assistance in molecular cloning. This work was supported by University and Research grant PRIN-2102 to F.C., by an ERA-NET-ANR Neuron grant ANR-15-NEUR-0002-04 to M.S, by "Investments for the Future" LabEx SIGNALIFE (grant ANR-11-LABX-0028-01) to M.S. and by a postdoctoral fellowship from the Ville de Nice, France ("Aide Individuelle aux Jeunes Chercheurs") to M.Bertacchi.

Received: January 29, 2018
Revised: August 2, 2018
Accepted: August 2, 2018
Published: August 30, 2018

REFERENCES

Alfano, C., and Studer, M. (2013). Neocortical arealization: evolution, mechanisms, and open questions. Dev. Neurobiol. *73*, 411–447.

Alfano, C., Magrinelli, E., Harb, K., Hevner, R.F., and Studer, M. (2014). Postmitotic control of sensory area specification during neocortical development. Nat. Commun. *5*, 5632.

Anand, S., Majeti, B.K., Acevedo, L.M., Murphy, E.A., Mukthavaram, R., Scheppke, L., Huang, M., Shields, D.J., Lindquist, J.N., Lapinski, P.E., et al. (2010). MicroRNA-132-mediated loss of p120RasGAP activates the endothelium to facilitate pathological angiogenesis. Nat. Med. *16*, 909–914.

Armentano, M., Chou, S.J., Srubek Tomassy, G., Leingärtner, A., O'Leary, D.D.M., and Studer, M. (2007). COUP-TFI regulates the balance of cortical patterning between frontal/motor and sensory areas. Nat. Neurosci. *10*, 1277–1286.

Assimacopoulos, S., Kao, T., Issa, N.P., and Grove, E.A. (2012). Fibroblast growth factor 8 organizes the neocortical area map and regulates sensory map topography. J. Neurosci. *32*, 7191–7201.

Barrett, S.D., Bridges, A.J., Dudley, D.T., Saltiel, A.R., Fergus, J.H., Flamme, C.M., Delaney, A.M., Kaufman, M., LePage, S., Leopold, W.R., et al. (2008). The discovery of the benzhydroxamate MEK inhibitors CI-1040 and PD 0325901. Bioorg. Med. Chem. Lett. *18*, 6501–6504.

Bertacchi, M., Parisot, J., and Studer, M. (2018). The pleiotropic transcriptional regulator COUP-TFI plays multiple roles in neural development and disease. Brain Res. https://doi.org/10.1016/j.brainres.2018.04.024.

Bertacchi, M., Pandolfini, L., Murenu, E., Viegi, A., Capsoni, S., Cellerino, A., Messina, A., Casarosa, S., and Cremisi, F. (2013). The positional identity of mouse ES cell-generated neurons is affected by BMP signaling. Cell. Mol. Life Sci. *70*, 1095–1111.

Bertacchi, M., Pandolfini, L., D'Onofrio, M., Brandi, R., and Cremisi, F. (2015). The double inhibition of endogenously produced BMP and Wnt factors synergistically triggers dorsal telencephalic differentiation of mouse ES cells. Dev. Neurobiol. *75*, 66–79.

Betel, D., Wilson, M., Gabow, A., Marks, D.S., and Sander, C. (2008). The microRNA.org resource: targets and expression. Nucleic Acids Res. *36*, D149–D153.

Borello, U., Madhavan, M., Vilinsky, I., Faedo, A., Pierani, A., Rubenstein, J., and Campbell, K. (2014). Sp8 and COUP-TF1 reciprocally regulate patterning and Fgf signaling in cortical progenitors. Cereb. Cortex *24*, 1409–1421.

Chiang, D.Y., Cuthbertson, D.W., Ruiz, F.R., Li, N., and Pereira, F.A. (2013). A coregulatory network of NR2F1 and microRNA-140. PLoS One *8*, 1–13.

Chiba, S., Kurokawa, M.S., Yoshikawa, H., Ikeda, R., Takeno, M., Tadokoro, M., Sekino, H., Hashimoto, T., and Suzuki, N. (2005). Noggin and basic FGF were implicated in forebrain fate and caudal fate, respectively, of the neural tube-like structures emerging in mouse ES cell culture. Exp. Brain Res. *163*, 86–99.

Chuang, J.-H., Tung, L.-C., and Lin, Y. (2015). Neural differentiation from embryonic stem cells in vitro: an overview of the signaling pathways. World J. Stem Cells *7*, 437–447.

Eendebak, R.J.A.H., Lucassen, P.J., and Fitzsimons, C.P. (2011). Nuclear receptors and microRNAs: who regulates the regulators in neural stem cells? FEBS Lett. *585*, 717–722.

Enright, A.J., John, B., Gaul, U., Tuschl, T., Sander, C., and Marks, D.S. (2003). MicroRNA targets in *Drosophila*. Genome Biol. *5*, R1.

Fukuchi-Shimogori, T., and Grove, E.A. (2001). Neocortex patterning by the secreted signaling molecule FGF8. Science *294*, 1071–1074.

Garel, S., Huffman, K.J., and Rubenstein, J.L. (2003). Molecular regionalization of the neocortex is disrupted in Fgf8 hypomorphic mutants. Development *130*, 1903–1914.

Greig, L.C., Woodworth, M.B., Galazo, M.J., Padmanabhan, H., and Macklis, J.D. (2013). Molecular logic of neocortical projection neuron specification, development and diversity. Nat. Rev. Neurosci. *14*, 755–769.

Hendrickx, M., Van, X.H., and Leyns, L. (2009). Anterior-posterior patterning of neural differentiated embryonic stem cells by canonical Wnts, Fgfs, Bmp4 and their respective antagonists. Dev. Growth Differ. *51*, 687–698.

Heng, J.I., Nguyen, L., Castro, D.S., Zimmer, C., Wildner, H., Armant, O., Skowronska-Krawczyk, D., Bedogni, F., Matter, J.M., Hevner, R., et al. (2008). Neurogenin 2 controls cortical neuron migration through regulation of Rnd2. Nature *455*, 114–118.

Hu, S., Wilson, K.D., Ghosh, Z., Han, L., Wang, Y., Lan, F., Ransohoff, K.J., Burridge, P., and Wu, J.C. (2013). MicroRNA-302 increases reprogramming efficiency via repression of NR2F2. Stem Cells *31*, 259–268.

Kang, I.H., Jeong, B.C., Hur, S.W., Choi, H., Choi, S.H., Ryu, J.H., Hwang, Y.C., and Koh, J.T. (2015). MicroRNA-302a stimulates osteoblastic differentiation by repressing COUP-TFII expression. J. Cell. Physiol. *230*, 911–921.

Leone, D.P., Srinivasan, K., Chen, B., Alcamo, E., and McConnell, S.K. (2008). The determination of projection neuron identity in the developing cerebral cortex. Curr. Opin. Neurobiol. *18*, 28–35.

Lichtfield, L.M., and Klinge, C.M. (2012). Multiple roles of COUP-TFII in cancer initiation and progression. J. Mol. Endocrinol. *49*, R135–R148.

Lupo, G., Bertacchi, M., Carucci, N., Augusti-Tocco, G., Biagioni, S., and Cremisi, F. (2014). From pluripotency to forebrain patterning: an in vitro journey astride embryonic stem cells. Cell. Mol. Life Sci. *71*, 2917–2930.

Naka-Kaneda, H., Nakamura, S., Igarashi, M., Aoi, H., Kanki, H., Tsuyama, J., Tsutsumi, S., Aburatani, H., Shimazaki, T., and Okano, H. (2014). The miR-17/106-p38 axis is a key regulator of the neurogenic-to-gliogenic transition in developing neural stem/progenitor cells. Proc. Natl. Acad. Sci. USA *111*, 1604–1609.

Qin, J., Tsai, S.Y., and Tsai, M.J. (2014). The critical role of COUP-TFII in tumor progression and metastasis. Cell Biosci. *4*, 58.

Robinson, M.D., McCarthy, D.J., and Smyth, G.K. (2010). edgeR: a Bioconductor package for differential expression analysis of digital gene expression data. Bioinformatics *26*, 139–140.

Rosa, A., and Brivanlou, A.H. (2011). A regulatory circuitry comprised of miR-302 and the transcription factors OCT4 and NR2F2 regulate human embryonic stem cell differentiation. EMBO J. *30*, 237–248.

Sansom, S.N., and Livesey, F.J. (2009). Gradients in the brain: the control of the development of form and function in the cerebral cortex. Cold Spring Harb. Perspect. Biol. *1*, a002519.

Sansom, S.N., Hébert, J.M., Thammongkol, U., Smith, J., Nisbet, G., Surani, M.A., McConnell, S.K., and Livesey, F.J. (2005). Genomic characterisation of a Fgf-regulated gradient-based neocortical protomap. Development *132*, 3947–3961.

Stenvang, J., Petri, A., Lindow, M., Obad, S., and Kauppinen, S. (2012). Inhibition of microRNA function by antimiR oligonucleotides. Science *3*, 1.

Storm, E.E. (2006). Dose-dependent functions of Fgf8 in regulating telencephalic patterning centers. Development *133*, 1831–1844.

Terrigno, M., Busti, I., Alia, C., Pietrasanta, M., Arisi, I., D'Onofrio, M., Caleo, M., and Cremisi, F. (2018). Neurons generated by mouse ES cell with hippocampal or cortical identity display distinct projection patterns when co-transplanted in the adult brain. Stem Cell Reports *10*, 1016–1029.

Toyoda, R., Assimacopoulos, S., Wilcoxon, J., Taylor, A., Feldman, P., Suzuki-Hirano, A., Shimogori, T., and Grove, E.A. (2010). FGF8 acts as a classic diffusible morphogen to pattern the neocortex. Development *137*, 3439–3448.

Van den Ameele, J., Tiberi, L., Vanderhaeghen, P., and Espuny-Camacho, I. (2014). Thinking out of the dish: what to learn about cortical development using pluripotent stem cells. Trends Neurosci. *37*, 334–342.

Wanet, A., Tacheny, A., Arnould, T., and Renard, P. (2012). miR-212/132 expression and functions: within and beyond the neuronal compartment. Nucleic Acids Res. *40*, 4742–4753.

Wang, W.-C., Lin, F.-M., Chang, W.-C., Lin, K.-Y., Huang, H.-D., and Lin, N.-S. (2009). miRExpress: analyzing high-throughput sequencing data for profiling microRNA expression. BMC Bioinformatics *10*, 328.

Wang, J., Park, J.W., Drissi, H., Wang, X., and Xu, R.H. (2014). Epigenetic regulation of miR-302 by JMJD1C inhibits neural differentiation of human embryonic stem cells. J. Biol. Chem. *289*, 2384–2395.

Yao, Z., Mich, J.K., Ku, S., Menon, V., Krostag, A.R., Martinez, R.A., Furchtgott, L., Mulholland, H., Bort, S., Fuqua, M.A., et al. (2017). A single-cell roadmap of lineage bifurcation in human ESC models of embryonic brain development. Cell Stem Cell *20*, 120–134.

Zufferey, R., Nagy, D., Mandel, R.J., Naldini, L., and Trono, D. (1997). Multiply attenuated lentiviral vector achieves efficient gene delivery in vivo. Nat. Biotechnol. *15*, 871–875.

Stem Cell Reports, Volume *11*

Supplemental Information

The microRNA *miR-21* Is a Mediator of FGF8 Action on Cortical COUP-TFI Translation

Marco Terrigno, Michele Bertacchi, Luca Pandolfini, Mario Baumgart, Mariantonietta Calvello, Alessandro Cellerino, Michèle Studer, and Federico Cremisi

SUPPLEMENTAL INFORMATION

SUPPLEMENTAL FIGURES

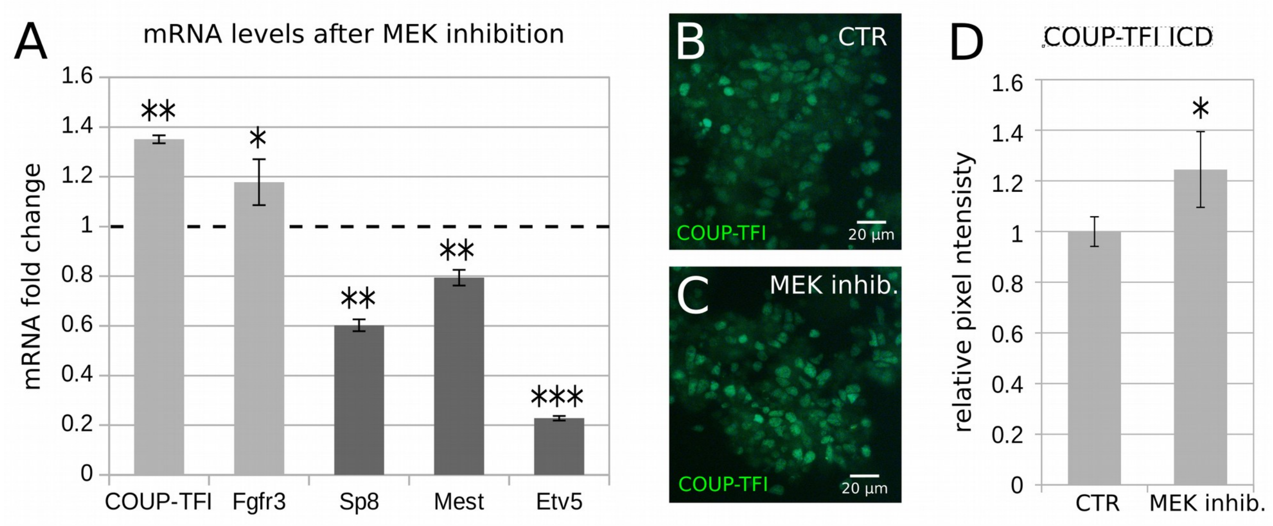

Figure S1 Effect of FGF signaling inhibition on A/P cortical markers and COUP-TFI protein

A, RT-PCR showing fold changes in *COUP-TFI, Fgfr3, Sp8, Mest* and *Etv5* transcript levels at DIV11, after 3 days of FGF signaling inhibition, obtained by means of the MEK inhibitor PD0325901. Error bars represent SEM (n = 3 independent experiments); * p≤0.05, ** p≤0.01, *** p≤0.001, Student T-test. **B-C**, COUP-TFI immunodetection in control DIV11 cells (B), and in DIV11 cells after 3 days of MEK inhibition. **D**, relative pixel intensity of COUP-TFI protein levels in control and MEK-inhibited cells as in B,C. N = 3 independent cultures. Error bars, SEM.

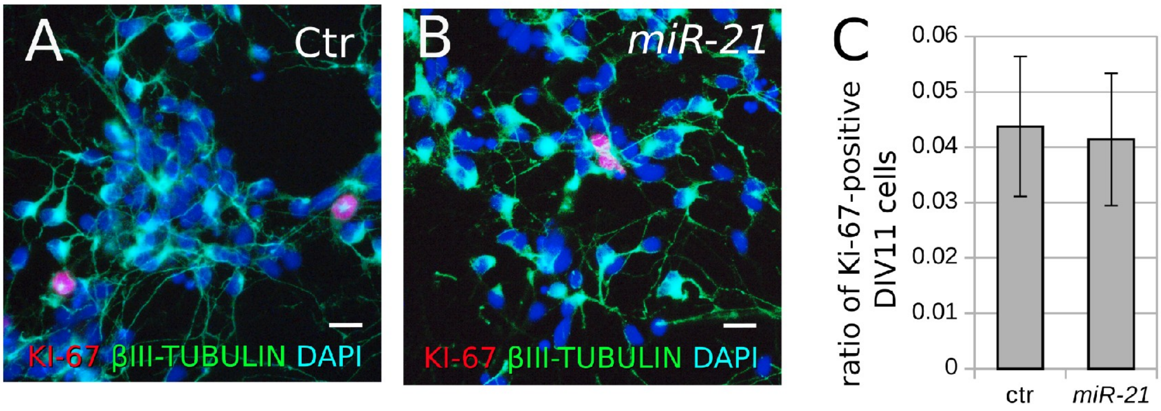

Figure S2 **Effect of *miR-21* upregulation on cell proliferation.**

A-B, KI-67 (red) and βIII-TUBULIN (green) immunodetection in DIV11 corticalized ES cells lipofected with scrambled (A, Ctr) or *miR-21* (B) mature miRNA at DIV8. Nuclei counterstaining (blue) was obtained with DAPI. Scale bars: 20 μm **C**, Quantification of KI-67- and βIII-TUBULIN-positive cell ratio. N = 3 independent cultures. Error bars, SEM.

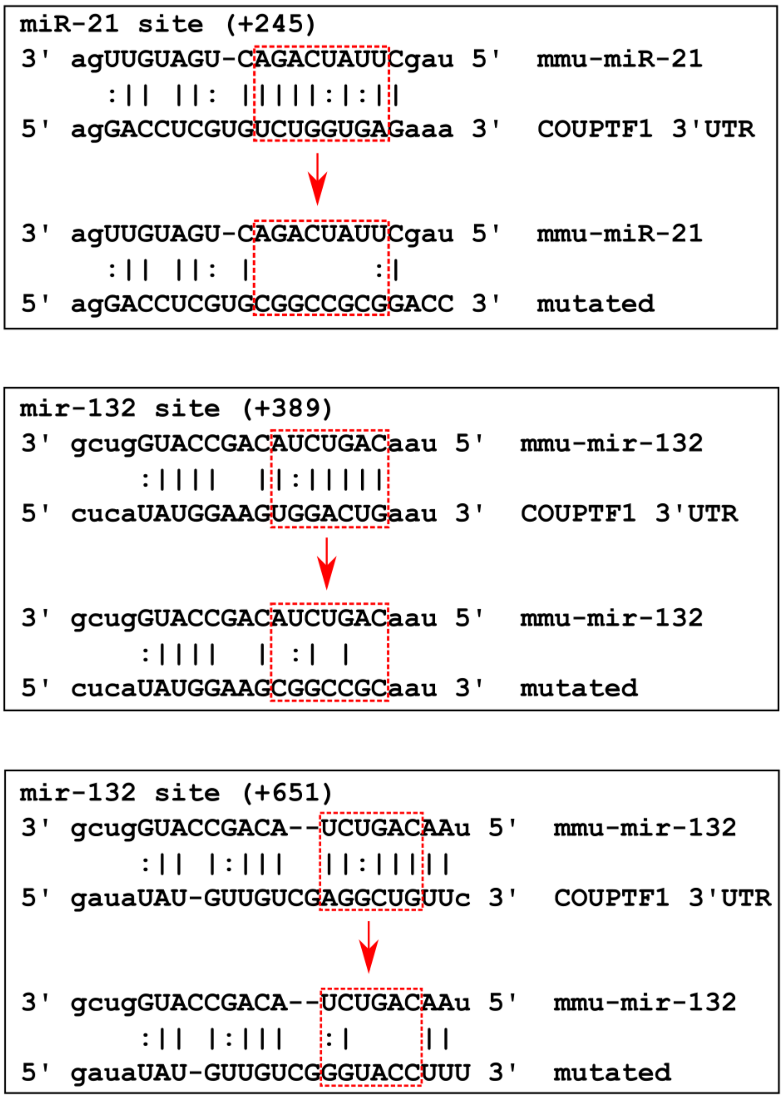

Figure S3 Mutagenesis of the predicted *miR-21* and *miR-132* binding sequences in *COUP-TFI* 3'UTR.

The dashed boxes indicate the seed sequence of miRNA/mRNA interaction predicted by miRANDA. Each seed sequence was mutated to generate distinct EGFP reporter mutants used in Fig. 5F (see Experimental Procedures).

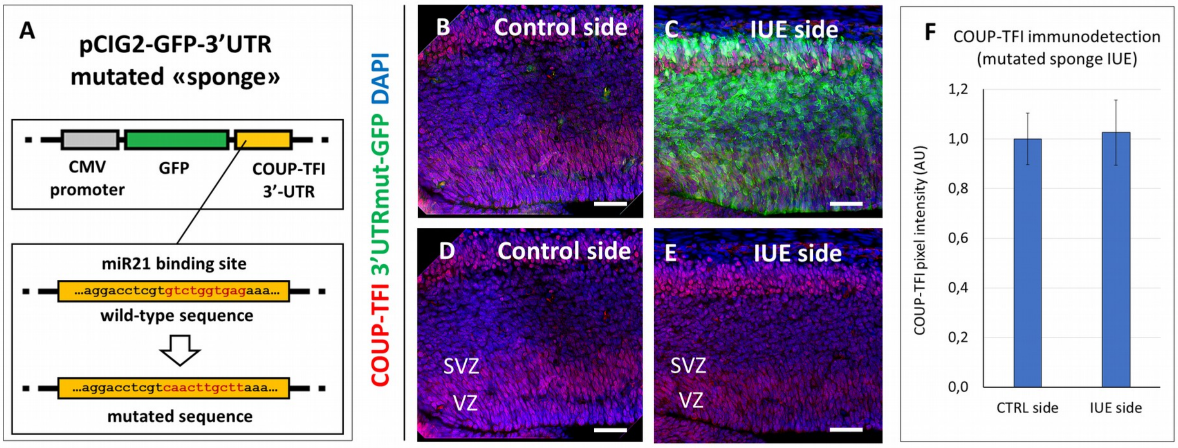

Figure S4 ***In vivo* IUE of *miR-21* seed-mutated sponge.**

A, Schematics of the control *in utero* electroporation (IUE) experiment using a mutated version of the *COUP-TFI* 3'UTR "sponge" plasmid. The seed sequence of the *miR-21* binding site (GTCTGGTGAG) was replaced with a neutral mutated sequence (CAACTTGCTT; nucleotides highlighted in red). The mutated "sponge" plasmid was injected into the lateral/dorsal pallium of one telencephalic hemisphere and electroporated in neural progenitors, as for experiment in Figure7. Brains were collected 48hr later and processed for immunofluorescence (IF). **B-E**, COUP-TFI IF (red) and GFP (green) in brain sections electroporated with the mutated "sponge" plasmid at E12.5 and analyzed at E14.5. The lateral/dorsal pallium of the electroporated hemisphere (C,E) was compared to the contralateral control hemisphere (B,D). Nuclei counterstaining (blue) was obtained with DAPI. Scale bars: 50 µm. **F**, Pixel intensity quantification (ImageJ) of COUP-TFI fluorescence in electroporated or control cortices (n = 3 brains). The staining was quantified only in the VZ and SVZ. Error bars, SEM.; there was not significant difference between the two conditions (t-test).

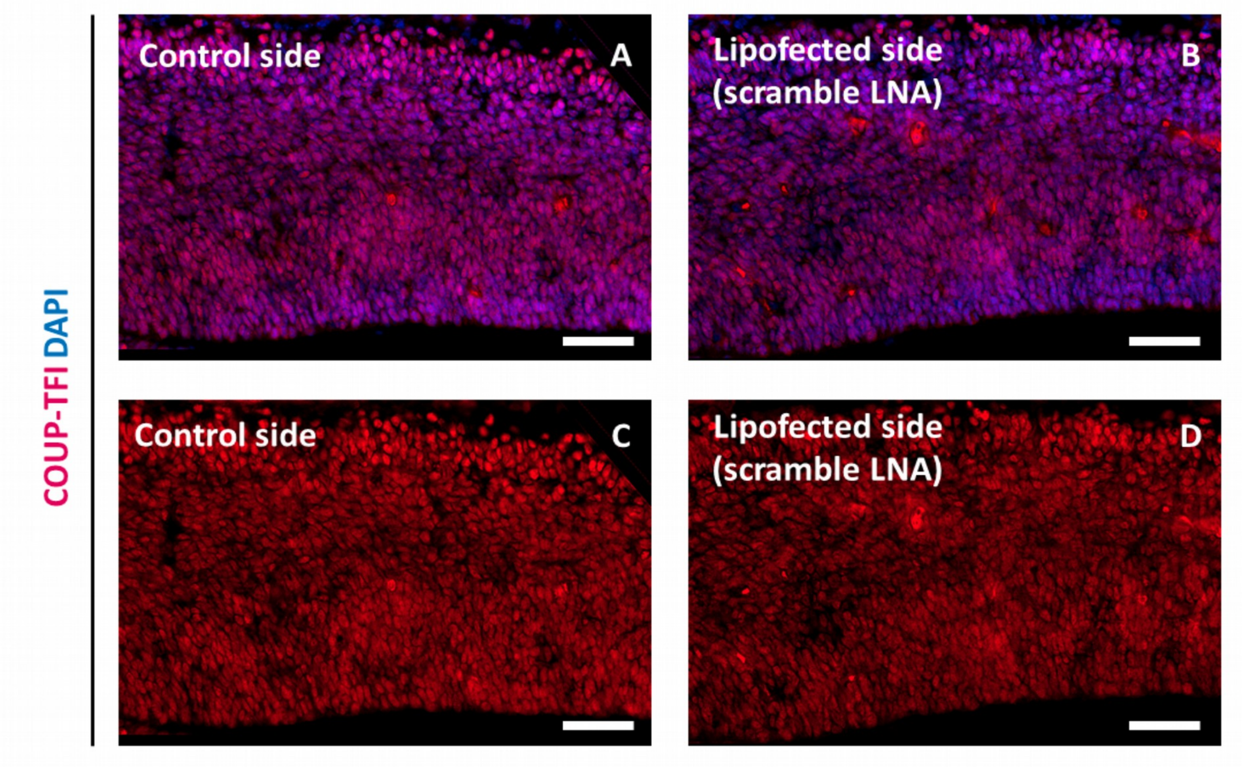

Figure S5 Control LNA lipofection *in vivo*

A-D, COUP-TFI IF (red) in E14.5 brain sections, 2 days after *in utero* lipofection of a scramble (control) LNA. The fluorescence intensity of COUP-TFI protein in neural progenitor regions of the lipofected hemisphere (B,D) was compared to the contralateral control (non-lipofected) hemisphere (A,C). Nuclei counterstaining (blue) was obtained with DAPI. The graph showing pixel intensity quantification corresponding to these images is displayed in Fig 7 L. Scale bar: 50 μm.

SUPPLEMENTAL EXPERIMENTAL PROCEDURES

ICD and RT-PCR

Cells prepared for immunocytodetection experiments were cultured on Poly-ornithine/Laminin coated round glass coverslips. Cells were fixed using 2% paraformaldehyde for 10-15 minutes, washed twice with PBS, permeabilized using 0.1% Triton X100 in PBS and blocked using 0.5% BSA in PBS. Primary antibodies used for ICD included CTIP2 (Abcam ab28448, 1:1000), SATB2 (Abcam ab51502, 1:400), COUP-TFI (Abcam ab60059, 1:1000), KI-67 (ThermoFisher PA5-16446, 1:1000) and TUJ1 (β-III TUBULIN, Covance MRB-435P, 1:1000). Alexa Fluor 488 and Alexa Fluor 546 anti-mouse or anti-rabbit IgG conjugates (ThermoFisher A-11001, A-11008, A-11030 and A-11010, all 1:500) were used as secondary antibodies. Primary antibodies were incubated 2hr at room temperature; cells were then washed three times with PBS (10' each). Alexa Fluor 488 and Alexa Fluor 546 secondary antibodies were incubated 1hr at RT in PBS containing 0.1% Triton X100 and 0.5% BSA for primary antibody detection, followed by three PBS washes (10' each). Nuclear staining was obtained with DAPI.

Total and small RNA was extracted with NucleoSpin RNA II or small RNA columns (Macherey-Nagel). RNA quantity and RNA quality were assessed with Nanodrop and gel electrophoresis. For each sample, 200ng of total RNA was reverse-transcribed, using Eurogentec cDNA synthesis kit for mRNAs and Qiagen miR-script kit for miRNAs. RT-PCR was performed using iTaq Universal SYBR Green Supermix (Bio-rad) on Rotor-Gene 6000 (Corbett). Amplification take-off values were evaluated using the built-in Rotor-Gene 6000 relative quantification analysis function, and relative expression was calculated with the $2^{-\Delta\Delta Ct}$ method, normalizing to the housekeeping gene *β-Actin* or *U6* for mRNAs or miRNAs, respectively. Standard errors were obtained from the error propagation formula, as described (Nordgård et al., 2006). N=3 independent cultures or brain explants were pooled together; each experiment contained n=2 technical replicates.

Reference

Nordgård O, Kvaløy JT, Farmen RK, Heikkilä R. Error propagation in relative real-time reverse transcription polymerase chain reaction quantification models: the balance between accuracy and precision. Anal Biochem. 2006, 356(2):182-93.

List of primers used for RT-PCR

**gene**	**forward sequence**	**reverse sequence**
beta-actin	AATCGTGCGTGACATCAAAG	AAGGAAGGCTGGAAAAGAGC
coupTFI	TGCTTGTGGCCTTGCGGATG	AGTTGCTCGATGACAGAGGAG
coupTFII	TCAACTGCCACTCGTACCTG	CCATGATGTTGTTAGGCTGCAT
efr1	ACGCGACACTACATTCCCC	CTCTTCCGTGTTACGAAGGGC
emx2	GGCTAGAGCACGCTTTTGAG	CACCGGTTAATGTGGTGTGT
en1	AGTGGCGGTGGTAGTGGA	CCTTCTCGTTCTTTTTCTTCTT
epha3	TTCTCCATCTCCGGTGAAAACA	ACCTCCCGACCAGAACATAGG
etv5	TCAGTCTGATAACTTGGTGCTTC	GGCTTCCTATCGTAGGCACAA
fgf15	ATGGCGAGAAAGTGGAACGG	CTGACACAGACTGGGATTGCT
fgf18	CCTGCACTTGCCTGTGTTTAC	TGCTTCCGACTCACATCATCT
fgf8	AGGGGAAGCTAATTGCCAAGA	CCTTGCGGGTAAAGGCCAT
fgfr3	GCCTGCGTGCTAGTGTTCT	TACCATCCTTAGCCCAGACCG
foxg1	CGACCCTGCCCTGTGAGT	TGGAAGAAGACCCCTGATTTTG
hey1	GCGCGGACGAGAATGGAAA	TCAGGTGATCCACAGTCATCTG
Id3	CTGTCGGAACGTAGCCTGG	GTGGTTCATGTCGTCCAAGAG
klf3	GAAGCCCAACAAATATGGGGT	GGACGGGAACTTCAGAGAGG
krox20	GCCAAGGCCGTAGACAAAATC	CCACTCCGTTCATCTGGTCA
mest	GTGGTGGGTCCAAGTAGGG	AAGCACAACTATCTCAGGGCT
otx2	CCACTTCGGGTATGGACTTG	GGTCTTGGCAAACAGAGCTT
pax6	CTTTGCTTGGGAAATCCGAG	AGCCAGGTTGCGAAGAACTC
PTN	ATGTCGTCCCAGCAATATCAGC	CCAAGATGAAAATCAATGCCAGG
sfrp1	CAACGTGGGCTACAAGAAGAT	GGCCAGTAGAAGCCGAAGAAC
sp8	CTGGTAGAGGGTAAGGGCGG	AAGATTGGAAAGGGGGTAGTGA
spry2	TCCAATGACGATGAGGACAA	CACCCCTGGCACAATTTAAG
